# Late Cretaceous crinoids (Echinodermata) from the southwestern margin of the Holy Cross Mts. (southern Poland) and phylogenetic relationships among bourgueticrinids

**DOI:** 10.1007/s12542-016-0313-9

**Published:** 2016-06-17

**Authors:** Rafał Lach, Mariusz A. Salamon

**Affiliations:** Department of Palaeontology and Stratigraphy, Faculty of Earth Sciences, University of Silesia, Będzińska 60, 41-200 Sosnowiec, Poland

**Keywords:** Crinoidea, Late Cretaceous, Holy Cross Mountains, Poland, Taxonomy, Phylogeny, Crinoidea, Oberkreide, Heiligkreuzgebirge, Polen, Taxonomie, Phylogenie

## Abstract

A systematic account of crinoids from the Upper Coniacian–Lower Campanian of the southwestern margin of the Holy Cross Mountains in southern Poland is presented. Seven crinoid taxa [*Marsupites testudinarius* (von Schlotheim), *Bourgueticrinus ellipticus* (Miller), *Bourgueticrinus* sp., *I*.? *granosus* Valette, *Isocrinus*? sp., *Nielsenicrinus carinatus* Roemer and *Austinocrinus bicoronatus* (von Hagenow)] are described and illustrated. The new material from Poland extends down the stratigraphic range of *Austinocrinus bicoronatus* to the Lower Campanian. Morphometric data support that *Bourgueticrinus ellipicus* and *B*.? *suedicus* are conspecific. Taphonomy and paleoecology of recorded crinoid assemblages are discussed. Phylogeny of Cretaceous bourgueticrinids is also revisited.

**Electronic supplementary material:**

The online version of this article (doi:10.1007/s12542-016-0313-9) contains supplementary material, which is available to authorized users.

## Kurzfassung

Es wird ein systematischer Überblick zu den oberkretazischen (Ober-Coniacium bis Unter-Campanium) Crinoiden vom SW-Rand des Heiligkreuzgebirges (Südpolen) vorgestellt. Insgesamt werden 7 Seelilientaxa, *Marsupites testudinarius* (von Schlotheim), *B. ellipticus* (Miller), *Bourgueticrinus* sp., *I.*? *granosus* Valette, *Isocrinus*? sp., *Nielsenicrinus carinatus* Roemer und *Austinocrinus bicoronatus* (von Hagenow), beschrieben und abgebildet. Die neuen Funde aus Polen erweitern die stratigraphische Reichweite von *Austinocrinus bicoronatus* bis in das frühe Campanium. Desweiteren implizieren die vorliegenden morphometrischen Daten, dass *Bourgueticrinus ellipicus* und *B.*? *suedicus* konspezifisch sind. Taphonomie und Paläoökologie der Crinoiden-Vergesellschaftung werden diskutiert, wie auch die Phylogenie der kreidezeitlichen Bourgueticriniden revidiert wird.

## Introduction

In recent years, major progress has been achieved through taxonomic studies on the Late Cretaceous crinoids from Poland (Salamon 2009; Salamon and Gorzelak 2010). Most recently, Lach (2016) completed and summarized our knowledge on this echinoderm class from this country. He also stressed that the Late Cretaceous crinoids are best known from the Vistula River Valley, Miechów Trough, Podlasie area, Upper Nysa Trough and Pomerania area (see also Niedźwiedzki and Salamon 2005; Salamon and Gorzelak 2010, 2011 and literature cited therein). A systematic account of Late Cretaceous crinoids from Opole, Roztocze, and the southwestern margin of the Holy Cross Mountains was also provided (see Appendix 1 in Lach 2016).

According to Remin (2004) and Lach (2016), the following crinoid taxa occur in the Upper Cretaceous sediments in the southwestern margin of the Holy Cross Mountains: *Isocrinus*? sp., *Bourgueticrinus* sp. (Coniacian); *Bourgueticrinus* sp., Comatulida indet., *Marsupites testudinarius* (von Schlotheim) (Santonian); Isocrinida indet. (Campanian). Thanks to the new findings of crinoid material from this region, we are able to provide herein a first detailed systematic description.

The material described in the present paper is housed at the Laboratory of Palaeontology and Stratigraphy of the University of Silesia (acronymed GIUS 9-3651).

## Geological framework

**Fig. 1:**
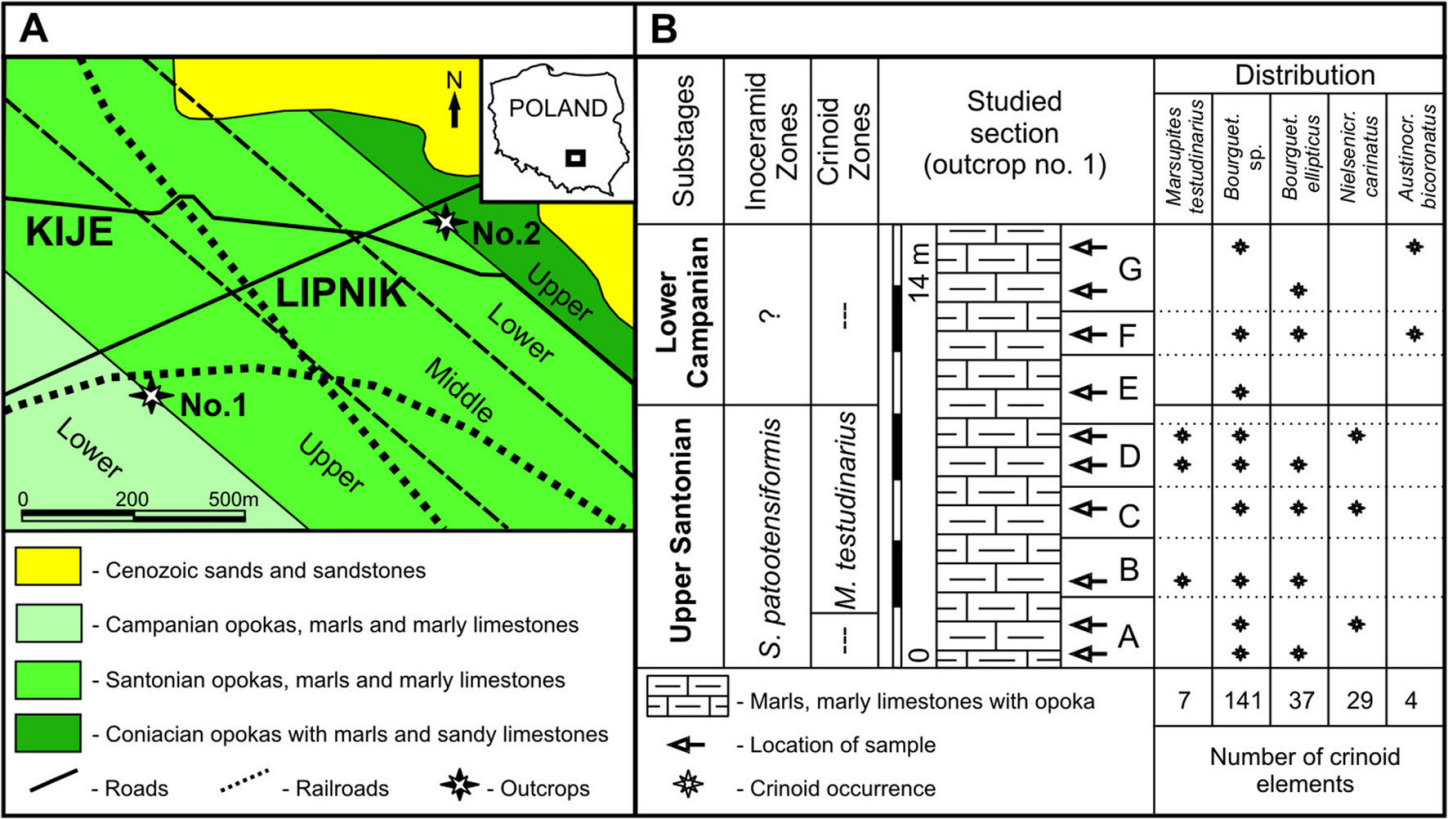
**a** Map of Poland with enlarged studied localities (slightly modified from Remin 2004, 2010). **b** Section of the outcrop no. 1. and distribution of collected crinoid taxa (modified after Remin 2010; biostratigraphy after Walaszczyk 1992)

The study area is located in the southwestern Mesozoic margin of the Holy Cross Mountains (HCM). This area is bordered by the so-called Szczecin–Łódź–Miechów Synclinorium to the south and the Mid-Polish Anticlinorium to the north. A number of outcrops of Upper Coniacian, Santonian, and Lower Campanian sediments are located near Kije and Lipnik villages. The so-called Lipnik–Kije depositional basin was a part of the Miechów Trough (Remin 2010; Fig. 1). Herein, a 150-m-thick, almost complete Santonian series is present (Remin 2010). However, according to Remin (2004, 2010), most of the outcrops are usually overgrown. A detailed description of the studied localities (Fig. 1) is given below (lithology and biostratigraphy from Remin 2004, 2010; Walaszczyk 1992).

*Outcrop no. 1* (Fig. 1a). Seven small outcrops (marked from A to G in Fig. 1b) located along the railway line were re-excavated during field works in 2013. 1.5–2 m-thick marly limestones and marls (commonly embedded with opoka) correspond to the Late Santonian and Early Campanian age. Sediments from the first outcrop (“A”) probably belong to the Late Santonian inoceramid *Sphenoceramus patootensiformis* Zone. The next outcrops (“B–D”, and partly “E”) represent the crinoid *Marsupites testudinarius* Zone. The outcrops referred to as “E”–“G” are probably Lower Campanian? in age as supported by the occurrence of ammonite *Gaudyceras mite* (details in Remin 2004, 2010).

*Outcrop no. 2* (Fig. 1a). Outcrop with Upper Coniacian and Lower Santonian sediments is located in the northern part of Kije village. The Upper Coniacian grey thick-bedded opoka (=calcarenite) corresponds to the ammonite *Texanites* *pseudotexanus* Zone and inoceramid *Sphenoceramus pachti* Zone and ranges up to the Early Santonian ammonite *Kitichnites emscheris/Nowakites savini* Zone. The upper part of the section with Lower Santonian sediments (opoka) corresponds to the inceramid *Cladoceramus undulatoplicatus* Zone (Walaszczyk 1992). According to Remin (2004, 2010), the total thickness of these sediments ranges from 20 to 25 m. However, only a small (~1 m) portion of this section is actually exposed. Herein, a small (~0.5-m-deep) pit was excavated. The occurrence of ammonite *Texanites* is indicative of a Coniacian age.

## Materials and methods

**Fig. 2:**
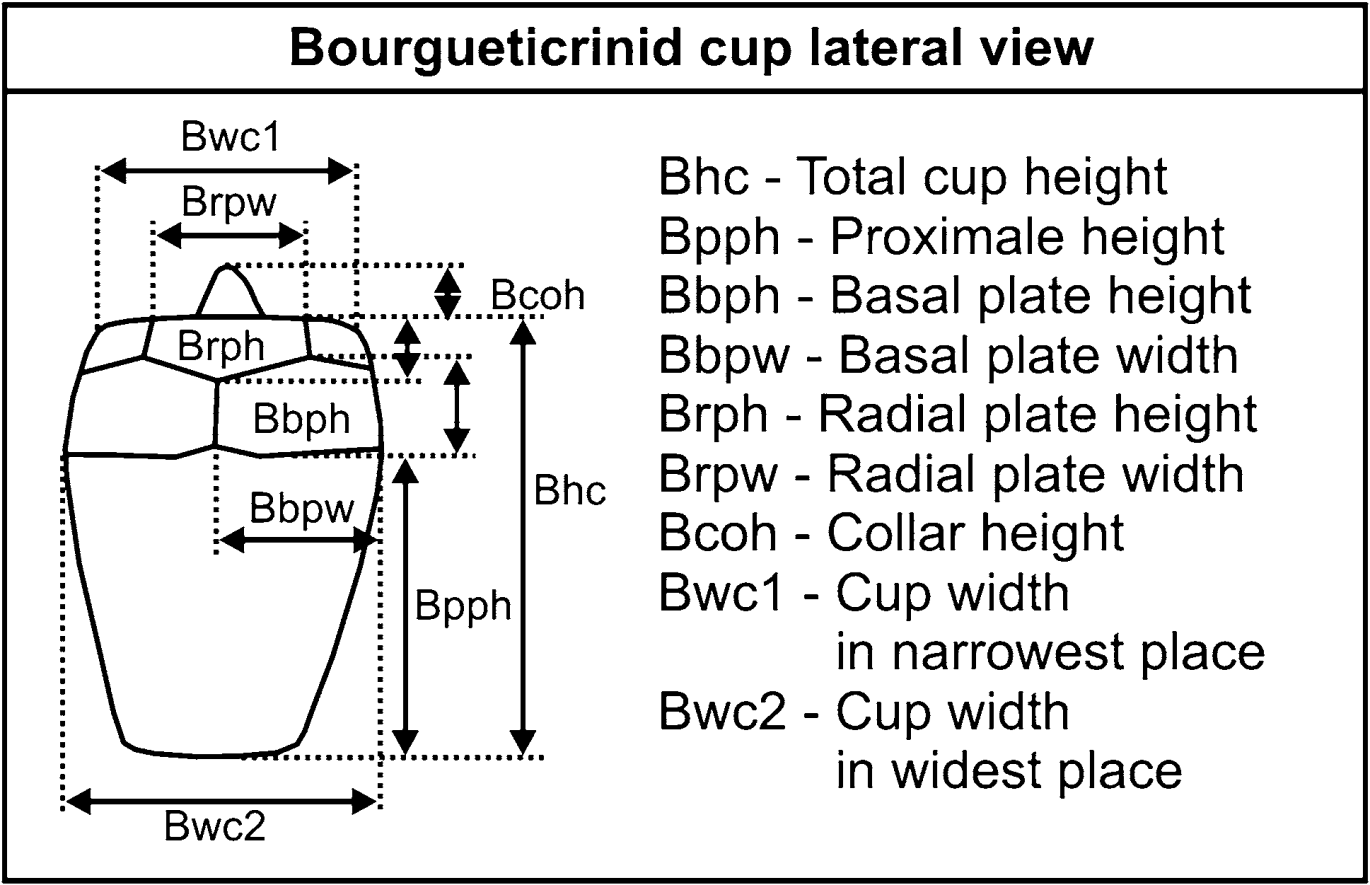
Dimensions of bourgueticrinid cups used in the present study (idea after Głuchowski 1987, modified)

During fieldwork (Spring and Autumn 2013), 10 rock samples of different ages (Santonian–Campanian), each weighing 10 kg, were gathered (outcrop no. 1; for details see Fig. 1). Additionally, three bulk samples (each weighing 10 kg) were taken from Coniacian sediments (outcrop no. 2). The samples were macerated with Glauber salt, washed with tap water, dried at 150 °C, and sieved through decreasing mesh widths (Ø 3.0, 1.5, 0.3 mm) following the work of Salamon et al. (2007). The echinoderm ossicles were then picked up under a stereoscopic microscope. Some specimens were additionally cleaned mechanically or by means of perchlorate. Nearly 750 crinoid ossicles (thecae, thecal plates, columnals, pluricolumnals, brachial plates, and cirrals) were collected (Fig 2).

In the Coniacian samples, besides dozens of crinoid ossicles, several ophiuroid, echinoid, and asteroid ossicles were also found. In the Santonian samples, numerous crinoid elements were collected along with abundant echinoid, ophiuroid, and asteroid ossicles. The Campanian samples delivered numerous crinoids and only a few echinoid and asteroid ossicles. Apart from echinoderms, bryozoans, gastropods, and inoceramid bivalves were also found in the Coniacian–Campanian samples. A Coniacian sample yielded a single ammonite fragment.

Biometric measurements were made using a stereoscopic microscope SM800T and electronic digital caliper (accuracy ±0.02 mm). Statistical analyses were performed using PAST software: PAlaeontological STatistics Version 1.94b (Hammer et al. 2001; for details see also Hammer and Harper 2006).

For comparison purposes, the Late Cretaceous crinoid collections (bourgueticrinids and marsupitids) housed at the Geologisch-Paläontologisches Museum in Hamburg (Gross Bülten and Lägerdorf collection, Hamburg, GPIH 4848-4850 and GPIH 40.1114/16-19, respectively), Museum für Naturkunde in Berlin (E.8411-8416), and at the Natural History Museum in London (Kent, Quidhampton and East Harnham near Salisbury collection, E45168-86 and E45320-6, respectively) were also examined.

## Systematic palaeontology

Systematics used in this paper follows Hess and Messing (2011). We adopted Rasmussen's (1961) isocrinid classification and terminology.

Class **Crinoidea** Miller 1821

Subclass **Articulata** Miller 1821

Order **Comatulida** A.H. Clark 1908

Superfamily **Uintacrinoidea** Zittel 1879

Family **Marsupitidae** d`Orbigny, 1852

Genus ***Marsupites*** Mantell in Miller 1821

*Type species* *Encrinites testudinarius* von Schlotheim 1820

*Diagnosis* The theca is composed of 16 plates grouped in three circlets of large and convex radial, basal, and infrabasal plates.

*Stratigraphic and geographic distribution* Upper Cretaceous (Late Santonian) of Africa (Algeria, Madagascar), Asia (Gulf Coast, India, Kazakhstan, Turkmenistan), Australia (Australia), Europe (England, France, Germany, Poland, Ukraine), Northern America (Canada, USA).

***Marsupites testudinarius*** (von Schlotheim 1820)

Figure 3a–j

**Fig. 3:**
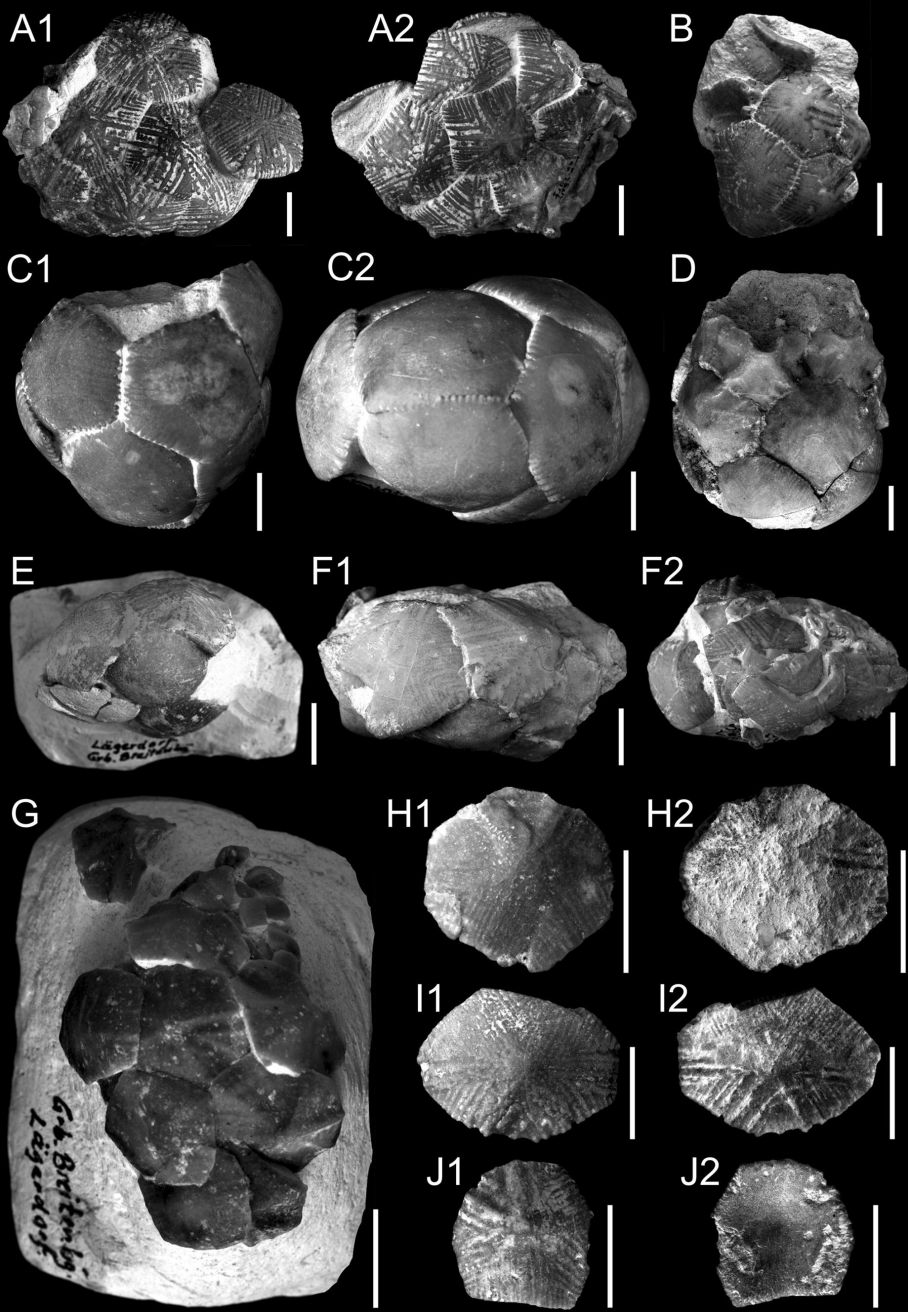
Santonian crinoid *Marsupites testudinarius* (von Schlotheim) from the southwestern margin of the Holy Cross Mountains and GPIH. *Scale* *bars* 1 cm. **a**–**g** Thecae. Upper Santonian. Lägerdorf. GPIH 40.1114/16-19. **h**–**j** Thecal plates. Upper Santonian. Lipnik–Kije Section. GIUS 9-3651/Mt/1-3

* 1820 *Encrinites testudinarius* von Schlotheim: p. 339.
- 1961 *Marsupites testudinarius* (von Schlotheim)—Rasmussen: p. 396–400, pl. 59, Figs. 11–17.
- 1994 *Marsupites testudinarius* (von Schlotheim)—Milsom et al.: p. 596, text-Fig. 1a.
- 2004 *Marsupites testudinarius* (von Schlotheim)—Remin: p. 593.
- v 2006 *Marsupites testudinarius* (von Schlotheim)—Łukowiak and Gorzelak: p. 784–786, Fig. 2a.
- v 2010 *Marsupites testudinarius* (von Schlotheim)—Salamon and Gorzelak: p. 15, Fig. 7d.
- v 2011 *Marsupites testudinarius* (von Schlotheim)—Salamon and Gorzelak: p. 313, Fig. 2i.

For a very detailed synonymy of *Marsupites testudinarius* see Lach (2016).

*Studied material* GIUS 9-3651/Mt: 7 thecal plates. GPIH 40.1114/16-19: 7 thecae, 15 brachials.

*Diagnosis* Theca is composed of three circlets of large and convex radial, basal, and infrabasal plates.

*Description* Thecal plates are of different sizes. They are mostly pentagonal or hexagonal in the case of basal plates. All plates are strongly convex. The plates with a maximum height of 21 mm are covered by 3–4 thick radiating ridges and between them numerous thin ridges are visible. Small plates with a minimum height of 13 mm are only covered by 3–4 thick radiating ridges. The inner surface of most plates is smooth. Only a few ossicles are covered by thin and long ridges on their inner surface. All thecal plates are thin (1.60–1.80 mm).

German specimens (GPIH): the thecae are of different sizes. Large specimens are spheroidal, ovoid, and strongly flattened. They consist of large plates with a maximum height of 22.5 mm, which are strongly ornamented by radiating ridges. The precise shape of the small thecae is difficult to ascertain because of compaction. These thecae are composed of small and smooth plates with a maximum height of 12.5 mm. The shape of IBr_1_ corresponds to the embayment in the radial plate. IBr_2_ are axilliaries. They are five-sided and possess upward-diverging sides. The articulation between IBr_1_ and IBr_2_ is syzygial. Few isolated brachial plates are secundibrachials; they are wedge-shaped and more or less angular; the plates are relatively low. Some of them are syzygial; others are muscular. The proximal, syzygial articulation is visible in a few specimens, and they have stout and radiating ridges. The pinnular sockets do not occur.

*Remarks* According to (Sieverts 1927; see also Brydone 1912 and Rasmussen 1961), two morphotypes of *Marsupites* can be distinguished. The lower part of the *Marsupites* zone is characterized by the presence of small and smooth thecae with only peripheral ornamentation of the plates (see also Łukowiak and Gorzelak 2006; Lach 2016). The upper part of the *Marsupites* zone is dominated by the presence of larger and strongly ornamented thecae. Rasmussen (1961) already mentioned that both morphotypes could co-occur. Furthermore, this latter synonymized many species of this genus and argued that the specimens described from the Campanian seem to be uncertain (see Rasmussen 1961, p. 400).

The material from Poland and Germany delivers both morphotypes. Though difficult to determine whether the thecal plates from Germany come from different stratigraphic intervals, we can only state with confidence that both morphotypes from Poland do not co-occur in the same layer, i.e., small and smooth plates were mostly found in the lower part of the section. Noteworthy is the fact that so far only large and strongly ornamented thecal plates have been recorded in Polish localities (details in Salamon and Gorzelak 2010, 2011; Lach 2016).

*Stratigraphic and geographic distribution*. Upper Cretaceous (Late Santonian) of Africa (Algeria, Madagascar), Asia (Gulf Coast, India, Kazakhstan, Turkmenistan), Australia (Australia), Europe (England, France, Germany, Poland, Ukraine), Northern America (Canada, USA).

Suborder **Bourgueticrinina** Sieverts-Doreck 1952

Family **Bourgueticrinidae** de Loriol 1882

Genus ***Bourgueticrinus*** d’Orbigny 1841

*Type species* *Apiocrinites ellipticus* Miller 1821

*Diagnosis* The theca with undivided proximale formed by one or several fused proximal columnals. Above the proximale occur a ring of basals and alternating with the basals a circle of radials.

*Remarks.* *Bourgueticrinus elegans* is the only bourgueticrinid that possesses no trace of basals.

*Stratigraphic and geographic distribution* Upper Cretaceous (Cenomanian)–Paleogene (Eocene) of Europe (Belgium, Denmark, England, France, Germany, Italy, Netherlands, Poland, Russia, Sweden, Ukraine), Northern America (USA).

***Bourgueticrinus ellipticus*** (Miller 1821)

Figures 4a–f, 5a–f

**Fig. 4:**
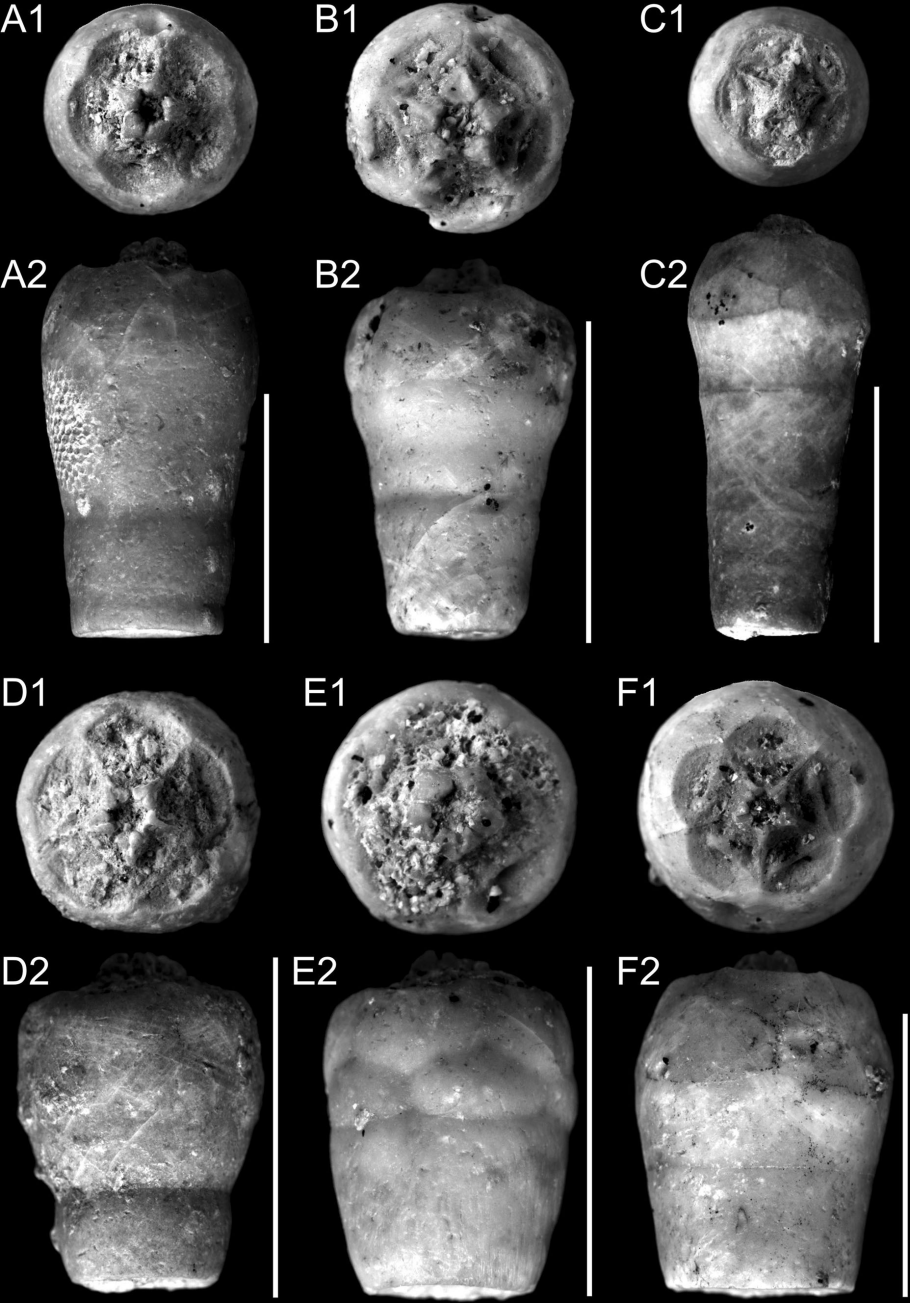
Upper Cretaceous crinoids from the southwestern margin of the Holy Cross Mountains. Upper Santonian/Lower Campanian. All from Lipnik–Kije Section. *Scale bars* 1 cm. A–F. *Bourgueticrinus ellipticus* (Miller). A1, A2 Thecae. Specimen with high collar and sloping articular faces. Upper and lateral views, respectively. GIUS 9-3651/Be/1. B1, B2 Thecae. Specimen with high collar and sloping articular faces. Upper and lateral views, respectively. GIUS 9-3651/Be/2. C1, C2 Thecae. Specimen with small collar and sloping articular faces. Upper and lateral views, respectively. GIUS 9-3651/Be/3. D1, D2 Thecae. Specimen with high collar and sloping articular faces. Upper and lateral views, respectively. GIUS 9-3651/Be/4. E1, E2 Thecae. Specimen with high collar and sloping articular faces. Upper and lateral views, respectively. GIUS 9-3651/Be/5. F1, F2 Thecae. Specimen with small collar and sloping articular faces. Upper and lateral views, respectively. GIUS 9-3651/Be/6

**Fig. 5:**
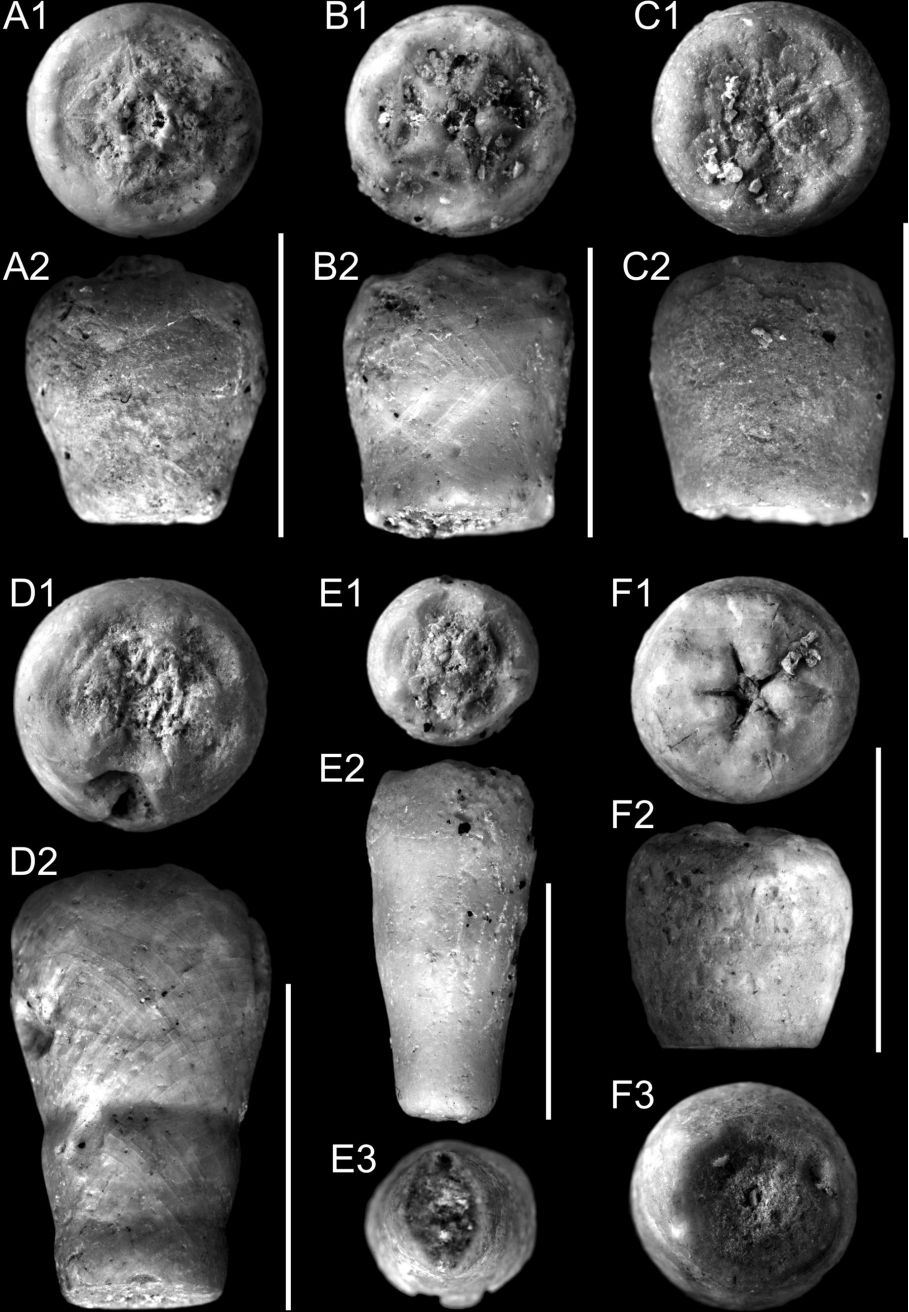
Upper Cretaceous crinoids from the southwestern margin of the Holy Cross Mountains. Upper Santonian/Lower Campanian. All from Lipnik–Kije Section. *Scale bars* 1 cm. **a**–**f**. *Bourgueticrinus ellipticus* (Miller). A1, A2 Thecae. Specimen with small collar and sloping articular faces. Upper and lateral views, respectively. GIUS 9-3651/Be/7. B1, B2 Thecae. Specimen with small collar and horizontal articular faces. Upper and lateral views, respectively. GIUS 9-3651/Be/8. C1, C2 Thecae. Specimen without collar and horizontal articular faces. Upper and lateral views, respectively. GIUS 9-3651/Be/9. D1, D2 Thecae. Specimen without collar and sloping articular faces. Upper and lateral views, respectively. GIUS 9-3651/Be/10. E1–E3 Thecae. Specimen with small collar, sloping articular faces, and elliptical cup base. Upper, lateral, and lower views, respectively. GIUS 9-3651/Be/11. F1–F3 Thecae. Specimen without collar, sloping articular faces, and circular cup base. Upper, lateral, and lower views, respectively. GIUS 9-3651/Be/12

* 1821 *Apiocrinites ellipticus*—Miller: p. 33, Figs. 1–7.
- 1841 *Bourgueticrinus ellipticus* Miller—Orbigny: p. 95, pl. 17, Figs. 1–6.
- 1848 *Bourgueticrinus milleri*—M’Coy: p. 405.
- 1850 *Apiocrinus ellipticus* Miller—Forbes in Dixon: p. 343, pl. 20, Figs. 12–16, 19, 25.
- 1881 *Mesocrinus suedicus*—Carpenter: p. 130, pl. 6, Figs. 3–7.
- 1917 *Volvola elliptica* (Miller)—Valette: p. 93, Fig. 3.
- 1961 *Bourgueticrinus ellipticus* (Miller)—Rasmussen: p. 182–184, pl. 24, Figs. 11–16, pl. 60, Fig. 2.
- 1961 *Bourgueticrinus suedicus* (Carpenter)—Rasmussen: p. 196–197, pl. 27, Fig. 1–8.
- 1982 *Bourgueticrinus ellipticus* (Miller)—Klikushin: p. 818, Fig. 4d, e, f.
- 1995 *Bourgueticrinus suedicus* (Carpenter)—Jagt: p. 191, Figs. 5, 7.
- 1999 *Bourgueticrinus*? *suedicus* (Carpenter)—Jagt: p. 129–130, pl. 31, Figs. 2–3, 5, 9, 11, ? pl. 36, Figs. 5–6, ?pl. 38, Figs. 8–9.
- v 2002 *Bourgueticrinus ellipticus* (Miller)—Smith and Wright: p.256, pl. 50, Fig. 6.
- v 2010 *Bourgueticrinus* cf. *ellipticus* (Miller)—Salamon and Gorzelak: p. 11–12, pl. 5a.
- v 2010 *Bourgueticrinus*? *suedicus* (Carpenter)—Salamon and Gorzelak: p. 14, pl. 4b.
- v 2011 *Bourgueticrinus*? *suedicus* (Carpenter)—Salamon and Gorzelak: p. 316, Fig. 3g.

*Studied material*. GIUS 9-3651/Be: 37 thecae, some of them with proximale and/or proximal columnals. GPIH 4848-4850: 453 thecae and proximale. MB E.8411-8416: 37 thecae. NHML E45168-86, E45320-6: 100 thecae.

*Dimensions* See supplementary materials.

*Diagnosis (emended)* Thecae are claviform, pyriform, conical, or pear-shaped. The diameter reaches its maximum at the top of the proximale or at the basals. The suture lines are distinct. The facets are horizontal or sloping outwards. The base of the proximale is elliptical or circular. The collar may be high, strongly reduced, or absent.

*Description* The thecae are smooth, large, claviform to pyriform, sometimes conical and pear-shaped. The basals and radials are five-sided and contact each other; they are usually of similar size; however, in some specimens they can be slightly higher than the others (see supplementary materials). The suture lines between proximale, basals, and radials are distinct; in some cases the suture lines are slightly depressed. The facets are lobate. The dorsal ligament fossae are concave and possess a shallow pit. The facets are horizontal, or slope more or less outwards and can form a high collar around the radial cavity; some collars are lower (1/4–1/2 of the height of the highest collars). The lateral ridges are relatively low and inconspicuous. The dorsal ligament fossae can be large and interarticular ligament fossae are distinctly smaller and separated by oblique ridges. The proximale, which constitutes the large part of the theca, is elliptical or circular in outline at the base. The largest theca diameter is observed at the top of the proximale or at the basals. The radial cavity is small or medium-sized. The columnals are subcylindrical with small and rounded lumen, and with synarthrial facets.

*Remarks*. Rasmussen (1961) noticed that *B*. *ellipticus* was very similar to *B*.? *suedicus* and *B. hureae* (Valette). He also has mentioned that the first two species were restricted to different stratigraphic levels, which strengthens their taxonomic separation. However, the stratigraphic ranges of both species seem to overlap, as recently suggested by several authors. Furthermore, morphometric data suggest that *B*.? *suedicus* and *B*. *ellipticus* are conspecific. More details can be found in the *Morphological variation of Bourgueticrinus* section.

*Stratigraphic and geographic distribution*: Upper Cretaceous (Lower Turonian–Upper Campanian) of Europe (Belgium, Czech Republic, England, France, Germany, Sweden, Poland, Russia, Ukraine), and Asia (Russia and its former Asian republics).

***Bourgueticrinus*** sp.

Figure 6a–c

**Fig. 6:**
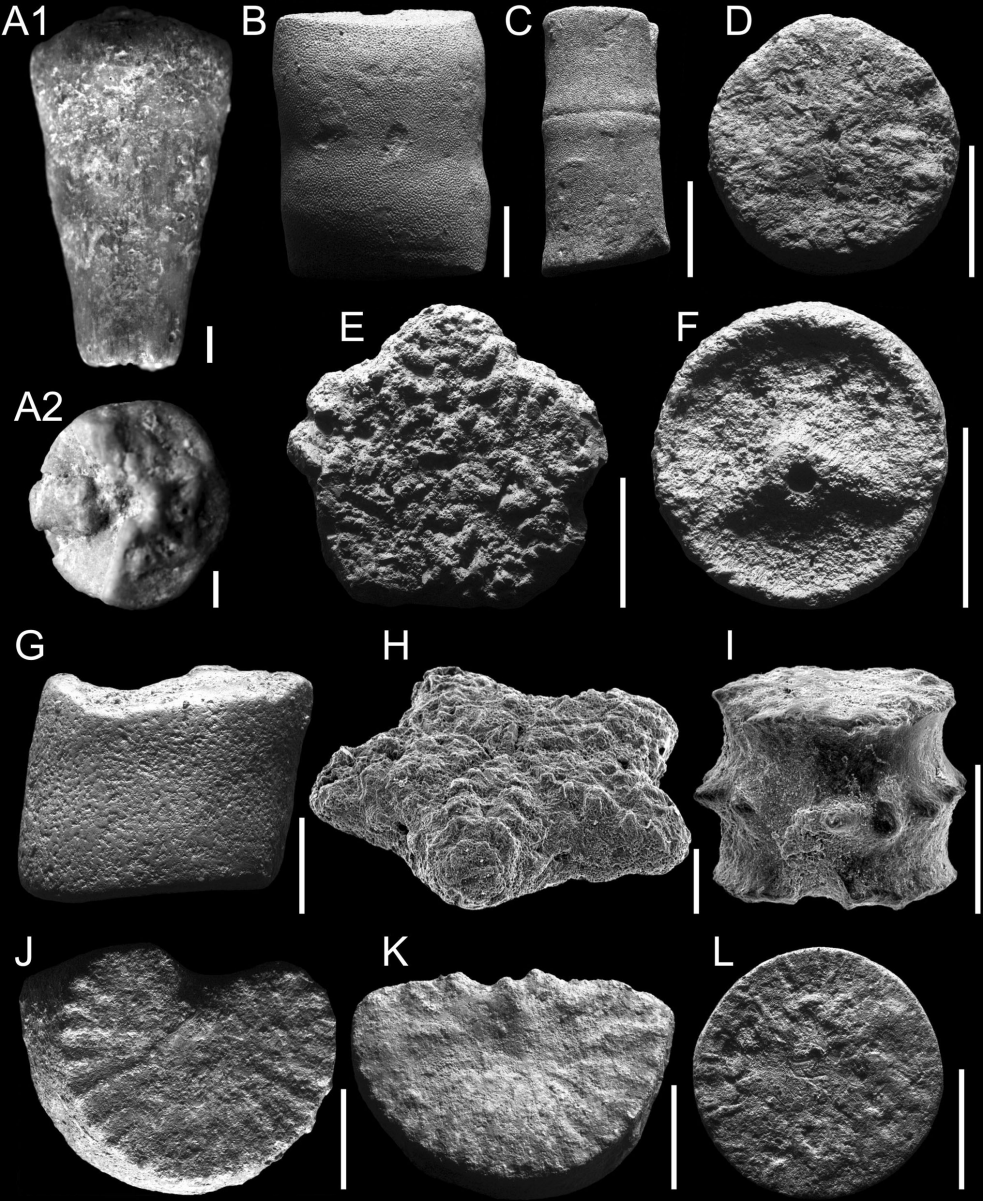
Late Cretaceous crinoids from the southwestern margin of the Holy Cross Mountains. All from Lipnik–Kije Section. *Scale bars* 1 mm. A–C *Bourgueticrinus* sp. A1, A2 Thecae. Lateral and upper views, respectively. Lower Campanian. GIUS 9-3651/B/3. B–C Columnals. Lateral view. Upper Coniacian. GIUS 9-3651/B/10-11. D *Isocrinus*? sp. Columnals. Articular face. Upper Coniacian. GIUS 9-3651/I/3. E–G *Isocrinus*? *granosus* Valette. E. Columnals. Articular face. Upper Coniacian. GIUS 9-3651/Ig/3. F, G Cirrals. Lateral and upper views, respectively. Upper Coniacian. GIUS 9-3651/Ig/11-12. H–K *Nielsenicrinus carinatus* Roemer. H–I. Columnals. Articular face and lateral views, respectively. Upper Santonian. GIUS 9-3651/Nc/1-2. J, K Brachials. Upper view. Upper Santonian. GIUS 9-3651/Nc/4-5. L *Austinocrinus bicoronatus* (von Hagenow). Columnals. Articular face. Lower Campanian. GIUS 9-3651/Ab/1

*Studied material.* GIUS 9-3651/B: 178 columnals, seven deformed thecae.

*Description* The columnals are of different sizes and shapes. Most of them are medium-sized, cylindrical, barrel-shaped, with elliptical ends that constricted medially. The facets are smooth or possess rhizocrinid pattern and fulcral ridge. The lateral surfaces are mainly smooth, planar, convex, or concave. The thecae are small, deformed, elongated, and thin. The sutures in a few cases are easily visible. The radial cavities are small and rounded.

*Remarks* Isolated bourgueticrinid columnals, which are strongly differentiated in shape and size, cannot be classified to the species-level. Similarly, the thecae at hand are small (juvenile?) and strongly deformed, which hinders their precise taxonomic affiliation (for comparison see Jagt and Salamon 2007; Salamon and Gorzelak 2010, 2011).

*Stratigraphic and geographic distribution.* Upper Cretaceous (Cenomanian)–Paleogene (Eocene) of Europe (Belgium, Denmark, England, France, Germany, Italy, Netherlands, Poland, Russia, Sweden, Ukraine), Northern America (USA).

Order **Isocrinida** Sieverts-Doreck 1952

Suborder **Isocrinina** Sieverts-Doreck 1952

Family **Isocrinidae** Gislén 1924

Subfamily **Isocrininae** Gislén 1924

Genus ***Isocrinus*** von Meyer in Agassiz 1836

*Type species*. *Isocrinites pendulus* von Meyer 1836

*Diagnosis* The species presents with a cup with small basals that are visible only from outside and not forming contiguous circlet. Identified as isocrinids with columnals that possess an elliptical petal floor surrounded by thin crenullae.

*Stratigraphic and geographic distribution* Triassic (?Carnian), Lower Jurassic—recent of the whole world.

***Isocrinus*** **?** ***granosus*** Valette 1917

Figure 6e–g

- ? 1850 *Pentacrinus* sp.—Dixon: p. 343, pl. 19, Fig. 2, pl. 20, Figs. 6, 7.
- 1961 *Isocrinus*? *granosus* Valette—Rasmussen: p. 130-133, pl. 16, Figs. 6–12, pl. 60, Fig. 1.
- 1992 *Isocrinus*? *granosus* Valette—Klikushin: p. 131.
- v 2010 *Isocrinus*? *granosus* Valette—Salamon and Gorzelak: p. 9, pl. 2A–D.

For very detailed synonymy of *Isocrinus*? *granosus* see Lach (2016).

*Studied material* GIUS 9-3651/Ig: 36 columnals (only internodal forms), 13 brachial plates, 45 cirrals?.

*Diagnosis* An isocrinid with articular face covered by granules or irregularities surrounding the lumen.

*Description* The columnals are (sub-)pentagonal to stellate in outline. The facet is covered by max. 18 long, thick, and rather short crenulae. The crenulae form granules or small irregularities around the lumen. The petal floors of moderate size are ellipsoidal in shape. The latera is covered by irregular tubercles located around the elevated, sharp keel. The lumen is circular and small. Brachial plates are rather small, V-shaped, and cryptosyzygial. The cirrals are wide and short, or moderately long. They are elliptical or rounded in section. The cirral facets are straight or slightly concave. The lumen is moderately large and raised above the perilumen. The cirral latera is smooth.

*Remarks*. Isocrinid columnals with granulated latera are commonly ascribed to *Isocrinus*? *granosus*. Rasmussen (1961) illustrated *I.*? *granosus* with petals possessing 20 crenulae. Similarly ornamented isocrinid species *I.*? *cenomanensis* (Orbigny) possesses slightly larger number of crenulae. Furthermore, the petals of the latter species are strongly elongated (compare to those in Valette 1917, Fig. 18; Rasmussen 1961, pl. 17, Figs. 4–8). Finally, this species is only known from the Albian and Cenomanian of western Europe. The cirrals at hand were also tentatively assigned to *I.*? *granosus*. According to Lach (2016; see also Salamon and Gorzelak 2010), cirrals possessing moderate or large lumen, rising above the perilumen, might belong to comatulids (Comatulida). However, comatulid centrodorsals were not found in the Coniacian sediments of investigated localities.

*Stratigraphic and geographic distribution* Cretaceous (Albian–Campanian) of Europe (Belgium, Czech Republic, Denmark, England, France, Germany, Netherlands, Poland, Sweden, Switzerland).

***Isocrinus*** **?** sp.

Figure 6d

*Studied material*. GIUS 9-3651/I: 290 columnals (252 internodals and 38 nodals), 11 pluricolumnals (up to two columnals), 87 brachials.

*Description* The columnals are small (with max. diameter up to 3 mm), pentagonal, pentalobate, or stellate in outline. The nodal columnals are larger than internodals. The facet is covered by max. 26 thin crenulae per petal in case of larger columnals. The facet of small specimens is covered by max. 18 stout crenulae per petal. The crenulae form granules around the lumen, especially in larger specimens. The marginal crenulae are rather thick and adradially fused V-like. The facets are covered by broad areolae of variable width. The petal floors are drop-like, sometimes ellipsoidal. The latera is smooth and straight. The cirrus scars of nodals are small and elliptical in outline with the articulum directed upwards. The aboral lip is sometimes present. The lumen is small and circular. The IBr_1_ are smooth and unornamented. The IBr_2_ is axillary. The articulation of IBr_1-2_ is syzygial.

*Remarks*. Fossil isocrinoids are mainly known from stalk fragments. Rasmussen (1961) proposed use of the provisional genus name ‘*Isocrinus*?’ for such isolated skeletal elements. Isolated isocrinid columnals with smooth latera were commonly described from the Upper Cretaceous of Poland as *Isocrinus*? sp. (e.g., Salamon and Gorzelak 2010, 2011, and literature cited therein). On the other hand, the columnals with ornamented latera were ascribed to *Isocrinus*? *granosus* Valette (Valette 1917; Rasmussen 1961; Salamon and Gorzelak 2010).

*Stratigraphic and geographic distribution* Triassic (?Carnian), Lower Jurassic—recent of the whole world.

Genus ***Nielsenicrinus*** Rasmussen 1961

*Type species*. *Pentacrinus obsoletus* Nielsen and Brünnich 1913.

*Diagnosis.* The isocrinid with radials and brachials that are coarsely granulate.

*Remarks* *Nielsenicrinus* is very similar to Cretaceous Isocrinidae but possesses a syzygial articulation; I Br_1–2_ is combined with a synarthial articulation II Br_1–2_.

*Stratigraphic and geographic distribution.* Lower Cretaceous (Hauterivian)–Paleogene (Oligocene) of Europe (Denmark, England, France, Germany, Netherlands, Sweden, Switzerland), Asia (Japan), and Australia (New Zealand).

***Nielsenicrinus carinatus*** (Roemer 1840)

Figure 6h–k

**Fig. 7:**
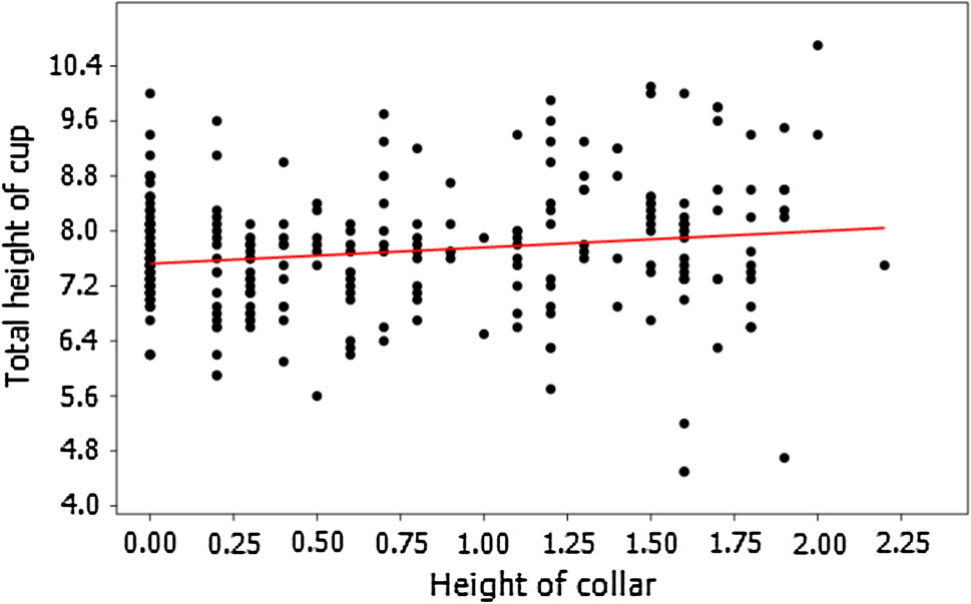
Relationship between the height of the collar and the total height of the cup (specimens from Geologisch-Paläontologisches Museum in Hamburg) showing strong positive correlation (Spearman's rank–order correlation coefficient *P* = 0.25022, *p* = 1.16E−05)

**Fig. 8:**
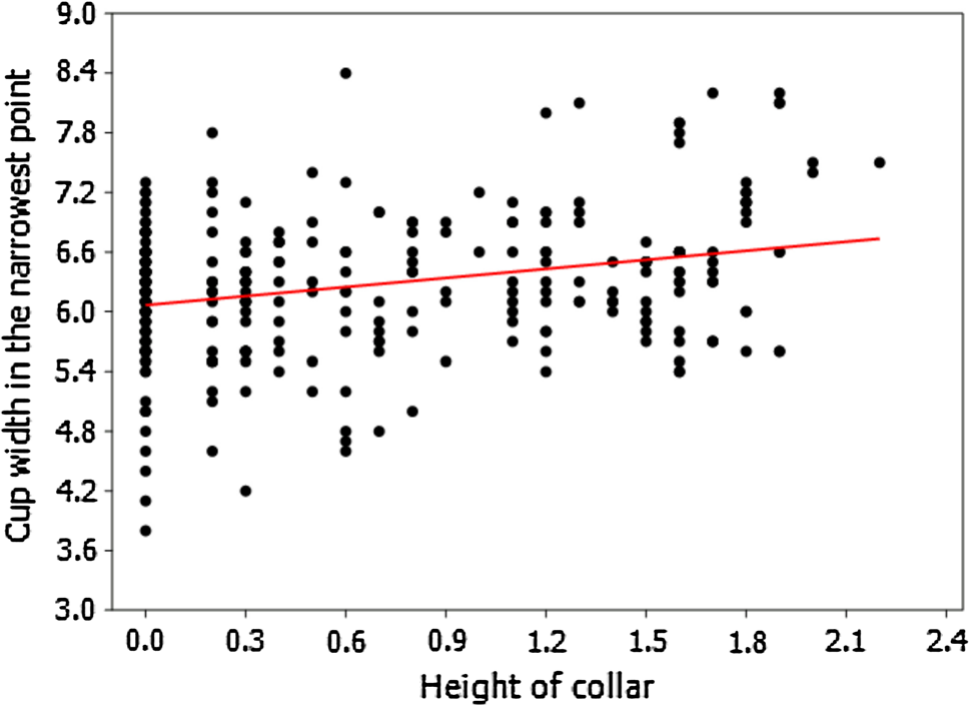
Relationship between the height of the collar and the cup width in the *narrowest point* (specimens from Geologisch-Paläontologisches Museum in Hamburg) showing strong positive correlation (Spearman's rank–order correlation coefficient *P* = 0.21836, *p* = 0.000138)

**Fig. 9:**
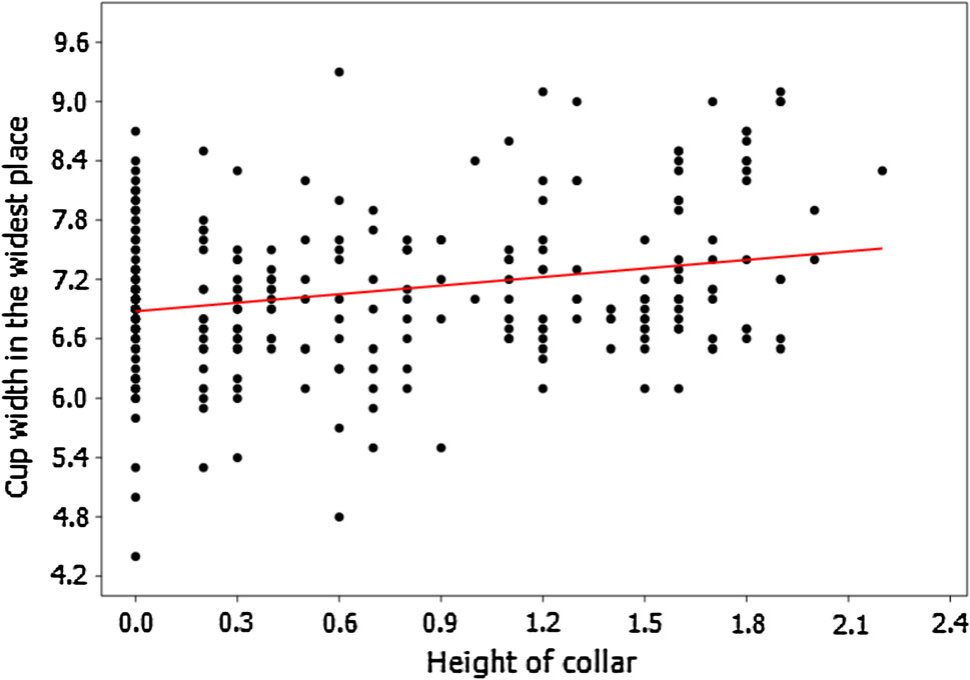
Relationship between the height of the collar and the cup width in the *widest point* (specimens from Geologisch-Paläontologisches Museum in Hamburg) showing strong positive correlation (Spearman's rank–order correlation coefficient *P* = 0.16179, *p* = 0.004969)

* 1840 *Pentacrinites carinatus*—Roemer: p. 26, pl. 6, Fig. 1.
- 1961 *Isocrinus*? *carinatus* (Roemer)—Rasmussen: p. 115, pl. 20, Figs. 3–8.
- 1961 *Isocrinus*? *minutus* (Valette)—Rasmussen: p. 141–143, pl. 15, Figs. 7–17.
- 1982 *Pentacrinus*? *carinatus* Roemer—Klikushin: p. 307.
- 1995 *Nielsenicrinus carinatus* (Roemer)—Jagt: p. 187, Figs. 3, 7.
- 1999 *Nielsenicrinus carinatus* (Roemer)—Jagt: p. 81–83; pl. 5, Figs. 4–7, 9–10, pl. 6, 7, 8 Figs. 1–9, ?10, 11; pl. 9–11; pl. 12, Figs. 1–6, 8.
- v 2011 *Isocrinus*? *minutus* (Valette)—Salamon and Gorzelak: p. 310–311, Fig. 2a.

For very detailed synonymy of *Nielsenicrinus carinatus* see Lach (2016).

*Studied material* GIUS 9-3651/Nc: 27 columnals (only internodals), two cryptosyzygial brachial plates.

*Diagnosis* Isocrinid with ‘spiny’ columnals and cryptosyzygial secundibrachials.

*Description* The columnals are circular to subpentagonal. The facets are covered by max. 16 crenulae. The marginal crenulae are clearly separated from the adradial crenulae. The adradial crenulae are sometimes reduced to granules. The lumen is small, circular. The latera is covered by granules or more commonly by small spines. The brachial plates, probably secundibrachials, are cryptosyzygial, relatively small, V- or U-shaped. The pinnular sockets are small, rounded, and deep.

*Remarks* The co-occurrence of cryptosyzygial brachials with ‘spiny’ isocrinid columnals suggests that these ossicles belong probably to *Nielsenicrinus carinatus* (Roemer). According to Oji et al. (1996), a diagnostic feature of nielsenicrinids is the presence of cryptosyzygies occurring between secundibrachials 3 and 4. Noteworthy is that (Salamon and Gorzelak 2010, but see also Jagt 1999) argued that Santonian ‘spiny’ species *I*.? *minutus* and Campanian *N*. *carinatus* are morphologically indistinguishable from each other.

*Stratigraphic and geographic distribution* Upper Cretaceous (Coniacian–Campanian) of Europe (Belgium, England, France, Germany, Netherlands, Poland, Russia, Ukraine), and Asia (Russia and its former Asian republics).

Genus ***Austinocrinus*** de Loriol 1889

*Type species*. *Austinocrinus komaroffi* de Loriol 1889 [=*Pentacrinus erckerti* Dames 1885].

*Diagnosis* Isocrinids with stout and very low circular and rounded columnals. The columnal facets possess a central ornament consisting of five interradial petals surrounded by very short and stout crenullae radiating from the petals. The crenullae are often replaced by adradial ridges. The marginal crenullae are often closely arranged or form isolated groups.

*Stratigraphic and geographic distribution* Upper Cretaceous (Early Campanian–Early Maastrichtian) of Africa (Tunisia), Asia (Caucasus, Turkmenistan), Europe (Belgium, Denmark, England, Germany, Netherlands, Poland, Spain, Sweden, Turkey), and South America (Mexico).

***Austinocrinus bicoronatus*** (von Hagenow 1840)

Figure 6l

* 1840 *Pentacrinus bicoronatus*—Hagenow: p. 663, pl. 9, Fig. 12.
- 1846 *Pentacrinus bicoronatus* Hagenow—Boll: p. 209.
- 1892 *Pentacrinus bicoronatus* Hagenow—Stolley: p. 249, 253, pl. 10, Figs. 2–6.
- 1904 *Pentacrinus bicoronatus* Hagenow—Jaekel: p. 195, Fig. 11.
- 1913 *Pentacrinus bicoronatus* Hagenow—Nielsen: p. 81.
- 1938 *Isocrinus* (*Pentacrinus*) *bicoronatus* (Hagenow)—Brydone: p. 4.
- 1961 *Austinocrinus bicoronatus* (Hagenow)—Rasmussen: p. 29-31, pl. 1, Figs. 1–9.
- v 1987 *Austinocrinus bicoronatus* (Hagenow)—Wright and Smith: p. 202, pl. 44, Figs. 1–2.
- 1992 *Austinocrinus bicoronatus* (Hagenow)—Klikushin: p. 118, 154, 160, 180.
- 1995 *Austinocrinus bicoronatus* (Hagenow)—Jagt: p. 186, Figs. 3, 7.
- 1999 *Austinocrinus bicoronatus* (Hagenow)—Jagt: p. 70–72, pl. 1, Figs. 1–2.
- v 2002 *Austinocrinus bicoronatus* (Hagenow)—Smith and Wright: p. 252, pl. 49, pl. 1, 2.
- v 2010 *Austinocrinus bicoronatus* (Hagenow)—Salamon and Gorzelak: p. 13, pl. 2 g.

*Studied material* GIUS 9-3651/Ab: four columnals (only internodals).

*Diagnosis* Isocrinid with articular faces that possess smooth and narrow areolae surrounded by very short crenulae.

*Description* The columnals are rounded to weakly subpentagonal. The facet is covered by smooth, rather narrow and lanceolate areolae that are bordered by short, V-shaped crenulae. The crenulae near the lumen are arranged in a form of granulae. The latera is smooth and straight. The lumen is smooth and circular.

*Remarks* Among six species of the genus *Austinocrinus* illustrated by Rasmussen (1961; *A. bicoronatus*, *A*. *cubensis* Valette, *A. erckerti* (Dames), *A. mexicanus* (Springer), *A. rothpletzi* Stolley, *A. solignaci* Valette), all are exclusively known from the Upper Cretaceous and Danian sediments (see also Klikushin 1975, 1982, 1983, 1985, 1992; Whittlesea 1991; Jagt 1999; Reich and Frenzel 2002; Reich et al. 2004). The first occurrence of this genus is in the Upper Campanian (with the exception of *A*. *rothpletzi*). Additionally, *A*. *cubensis*, *A*. *mexicanus*, and *A*. *solignaci* were documented only in Africa and northern America. Jagt (1999) reminded us that *A rothpletzi* appears to be typical for the Late Campanian, although some other authors reported this species from the Santonian (e.g., Klikushin 1983). The latter species differs from *A*. *bicoronatus* in having a specific petaloid structure. Furthermore, in contrast to *A*. *bicoronatus*, the columnals of *A*. *rothpletzi* are commonly pentagonal and (sub-)stellate and the marginal crenelae are distinct, long, and thin (compare Stolley 1892, pl. 10, Figs. 7–14; Jaekel 1904, Figs. 1–10; Sieverts-Doreck 1952, pl. 17, Figs. 1–13, pl. 18, Figs. 1–7, 12, text-Figs. 1–6; Rasmussen 1961, pl. 2, Figs. 1–9). These features allowed the assignment of the material at hand to *A*. *bicoronatus*. Jagt (1999) pointed out that the transitional forms are typical of the Campanian/Maastrichtian boundary. It is likely that the present form is also transitional between older *A*. *rothpletzi* and younger *A*. *bicoronatus*. On the other hand, it cannot be excluded that the age of the sediments in HCM was incorrectly established [the datation is only supported by the single specimen of ammonite *Gaudryceras* *mite* (von Hauer) (Remin 2004)].

Klikushin (1975, 1992) updated the species list of the genus *Austinocrinus* by: (1) *A. albaticus* Klikushin = *A. komaroffi* and *A. komarovi* (Coniacian–Santonian; Crimea), (2) *A. turkmenicus* Klikushin (Santonian–Campanian). Klikushin (1975) also erected a new species from Turkmenistan, but in his monograph from 1992 he suggested that this new species should be assigned to *A. rothpletzi* (see also Jagt 1999). Donovan et al. (1994) also recorded *Austinocrinus* n.sp. from the Aptian of Jamaica, but further on they assigned their material to millericrinid crinoid *Apiocrinites* sp (Donovan et al. 1996).

*Stratigraphic and geographic distribution* Upper Cretaceous (Early Campanian–Early Maastrichtian) of Europe (Belgium, Denmark, England, Germany, Netherlands, Poland, Sweden).

## Taphonomy

Taphonomic analyses of the material at hand indicate a state of preservation similar to those of other crinoid assemblages described so far from elsewhere in Poland (Jagt and Salamon 2007; Salamon et al. 2007, 2009; Salamon and Gorzelak 2010, 2011; Lach 2016). The majority of the crinoids are represented by isolated ossicles, although articulated cups and pluricolumnals are also present. The observed pattern of disarticulation and low frequency of abrasion or secondary alteration of ossicle shape (cf. Gorzelak and Salamon 2013; Salamon et al. 2014) suggest that after the death of these crinoids, their skeletons were not transported over considerable distance but probably stayed for a longer time at the sediment–water interface before final burial. Dissolution traces, evidence of mineral coating, and bioerosion are rarely observed in the ossicles at hand. Epibionts were observed on 21 % of ossicles. They are represented by bryozoans assignable to Cyclostomata (“*Berenicea*” and *Stomatopora*), Cheilostomata (Calloporidae), as well as formaninifers (*Bullopora*?). Deformations of stereom (so-called swellings) were not observed, suggesting a post-mortem incrustation. Furthermore, 19 % of ossicles bear various scratches and pits on the latera. They are mostly thin and slightly elongated (up to about a few mm). Such traces were commonly interpreted as bite marks produced by predatory sea urchins (Gorzelak and Salamon 2009; Salamon and Gorzelak 2010; Baumiller et al. 2010; Gorzelak et al. 2012). It is noteworthy that Salamon and Gorzelak (2010) recorded a similar frequency (24 %) of bite marks in the Late Cretaceous crinoids from the Middle Vistula River Valley.

## Paleoecology

Recorded crinoid assemblages are dominated by benthic forms. Bourgueticrinid crinoids were sessile forms permanently attached to the seafloor by radicular cirri. On the other hand, isocrinoids are considered as motile benthic forms capable of movement on the seafloor with arms (Baumiller and Messing 2007). The only stalkless crinoid recorded in the Upper Cretaceous of the Holy Cross Mountains is the comatulid species *Marsupites testudinarius*. The mode of life of these crinoids has been the source of considerable controversy. According to Hess and Messing (2011), *Marsupites* Mantell in Miller and *Uintacrinus* Grinnel are very similar to each other, and both are gathered into the superfamily Uintacrinoidea. Thecae of these crinoids are bowl-shaped, composed of thin plates displaying extremely long arms (e.g., Hess 1999). Representatives of the genus *Marsupites* are mostly known from the Santonian (e.g., von Schlotheim 1820; Miller 1821; Springer 1911; Sieverts 1927). Sieverts (1927) and Rasmussen (1961) stated that the different species of *Marsupites* are indeed a single species, *M*. *testudinarius*. Apart from Santonian occurrences, these crinoids were also recorded in the Campanian of Madagascar (Besairie 1936). This latter author documented very large specimens, which are now stored in the British Museum. However, Rasmussen (1961) mentioned that “this isolated, late stratigraphical occurrence of a *Marsupites* seems uncertain and may be incorrect”. Representatives of the genus *Uintacrinus* (three species) are exclusively known from the Santonian. Although *U*. *socialis* Grinnel was based on complete specimens, *U*. *anglicus* (Brydone) is only known from isolated material. Rasmussen (1961) mentioned that the latter species differs from *U*. *socialis* by having the wrinkled surface of the radials and proximal brachials. The third species *U. westfalicus* Schlüter was synonymized by Rasmussen (1961) with *U. socialis*. Although Schlüter (1878) specified many morphological differences between both species, Springer (1901) argued that they are difficult to verify.

As mentioned above, much attention has been paid to the uintacrinid mode of life. The first interesting hypothesis was provided by Bather (1889). According to the latter author these crinoids were swimming forms possessing five arms raised upward and five others directed downward. Springer (1901) mentioned that these crinoids were not capable of swimming but rather lived in groups on the soft sea bottom. Somewhat later, Kirk (1911) suggested that they might be gregarious species that were swimming in some sort of a shoal towards the shallow sea for spawning purposes. Jaekel (1918) did not agree with the “swimming” hypothesis and argued that their skeletons were probably too massive. This latter source stated that these crinoids rested on the seafloor with broadly expanded arms. Abel (1927) assumed that *U. socialis* displayed a planktonic mode of life. Hyman (1955) hypothesized that these forms were able to swim with matted arms directed downward. Struve (1957) provided an interesting hypothesis suggesting that *Unitacrinus* displayed a mode of life akin to that of ophiuroids and asteroids. This latter suggested that the mouth of these animals was directed toward the bottom and that they were crawling like ophiuroids. In accordance with Hyman’s (1955) hypothesis, Breimer and Lane (1978) argued that uintacrinids were planktonic forms with arms directed toward the bottom. These latter suggested that the thin thecae of these animals were filled with gas or oil. More recently, Milsom et al. (1994) postulated that these crinoids were benthic forms whose thecae were embedded in the sediment. The proximal part of their arms might have lain on the seafloor whereas the more distal portions might have been positioned upright, building a feeding bowl. In accordance, Hess (1999) argued that uintacrinids were benthic forms and their widespread paleogeographic distribution might reflect a planktonic juvenile stage of unusual duration among crinoids. Based on the functional morphology of arms and thecae, Seilacher and Hauff (2004) suggested that *Uintacrinus* was a hemipelagic dredger. These latter shared the previous hypothesis from Breimer and Lane (1978) that the thecae of these crinoids were filled with gas. Additionally, they argued that the distal portions of the arms were directed downward and were trailing on the seafloor, so that these animals could have been genuine deposit feeders. Seilacher and Hauff (2004) stressed that the taphonomic data were not in conflict with their idea. During storms, their arms likely tangled with each other, causing a buoyancy decrease and their fall to the seafloor. However, in our view the hemipelagic dredger hypothesis seems to be highly unlikely, as the filtration fans of these crinoids might have been particularly prone to clogging with sediment particles. Thus, the benthic lifestyle postulated by Milsom et al. (1994) and Hess (1999) is the most probable.

## Morphological variation of *Bourgueticrinus ellipticus*

As mentioned above, the cups of *B*.? *suedicus* are morphologically similar to those of *B*. *ellipticus*. According to Rasmussen (1961), *B*.? *suedicus* and *B. ellipticus* display different stratigraphic ranges that justify their taxonomic separation. On the other hand, Rasmussen, when describing *B. ellipticus* and *B*.? *suedicus*, mentioned that these species are restricted to Turonian?, Coniacian?–Campanian, and Santonian–Campanian, respectively. The scheme (Rasmussen 1961, p. 412) indicates that these two species may co-occur (review in Table 1 in Jagt and Salamon 2007). With respect to the morphological differences between *B. ellipticus* and *B*.? *suedicus*, it has been argued that: (1) *B*.? *suedicus* displays high collar on the theca, which is not observed in *B. ellipticus*; (2) in contrast to *B*.? *suedicus*, *B*. *ellipticus* displays almost horizontal facets; (3) the bases of the cups in *B*.? *suedicus* and *B*. *ellipticus* are elliptical and circular, respectively (compiled after Valette 1917; Rasmussen 1961; Klikushin 1982; Salamon and Gorzelak 2010). Rasmussen (1961) highlighted that the major diagnostic feature distinguishing both species is the presence of almost horizontal articular facets in *B. ellipticus*, whereas they are sloping in *B*.? *suedicus*.

In the present paper, a large collection of cups attributed to *B*.? *suedicus* and *B. ellipticus* from the Santonian of Germany (GPIH and MB), England (NHML), and Poland (Holy Cross Mountains) was investigated. It appeared that both species display a wide range of morphological variation. In particular, the inclination of the radial articular facet varies significantly, i.e., there are a number of ‘transitional’ forms. Furthermore, the height of the collar as well as the shape of the cup base vary significantly in these specimens (see Supplementary Appendix 1). For example, among 300 bourgueticrinid cups investigated from the Santonian collection of Hamburg, 107 specimens possess horizontal articular facets, among which 34.6 % display an elliptical cup base. Furthermore, 52.3 % of the specimens with horizontal articular facets yield a collar. The number of ‘transitional’ forms possessing slightly sloping articular facets is also large (96 specimens). Among these ‘transitional’ forms, the frequency of specimens displaying circular (44.8 %) and elliptical (55.2 %) cup bases is comparable. Also, the frequency of specimens with and without a collar does not differ significantly (75 and 25 %, respectively). The number of specimens with strongly sloping articular facets (97 specimens) is comparable to that of ‘transitional’ specimens as well as to those with horizontal articular facets. Among the forms with strongly sloping articular facets, the frequency of specimens with a circular cup base is high (34 %). Similarly, the frequency of specimens without a collar is significant (25.8 %).

To further test the reliability of separation between these two species, detailed biometric analyses were conducted. For this purpose, the collection from the Geologisch-Paläontologisches Museum in Hamburg appeared the most suitable since it is rich in well-preserved bourgueticrinid cups. Various morphological characters were measured (details in Supplementary Appendix 1). Biometric data of their cups revealed that they are not clustered into two morphologically distinct groups. For example, the height of the collar correlates positively with the total height of the cup (Spearman's rank–order correlation coefficient *P* = 0.25022, *p* = 1.16E−05; Fig. 7) and with the cup width (both in the narrowest (Spearman's rank–order correlation coefficient *P* = 0.21836, *p* = 0.000138; Fig. 8) and in the widest point (Spearman's rank–order correlation coefficient *P* = 0.16179, *p* = 0.004969; Fig. 9). Furthermore, after splitting these biometric data into two groups according to diagnosis (i.e., the first group comprising the forms with strongly sloping articular faces and the second one with slightly sloping or with horizontal articular faces), it appeared that they cannot be statistically segregated from each other. The non-parametric Mann–Whitney tests report a probability of *p* ≫ 0.05 for equality of medians of the total cup height and width, proximale height, basal height and width, radial height and width, giving off non-significant differences in any case also after Bonferroni corrections. These data strongly support that *B*.? *suedicus* appears to comprise variants or ecophenotypes of a coeval, single species, *B*. *ellipticus*.

## Phylogeny of Cretaceous bourgueticrinid species

**Fig. 10:**
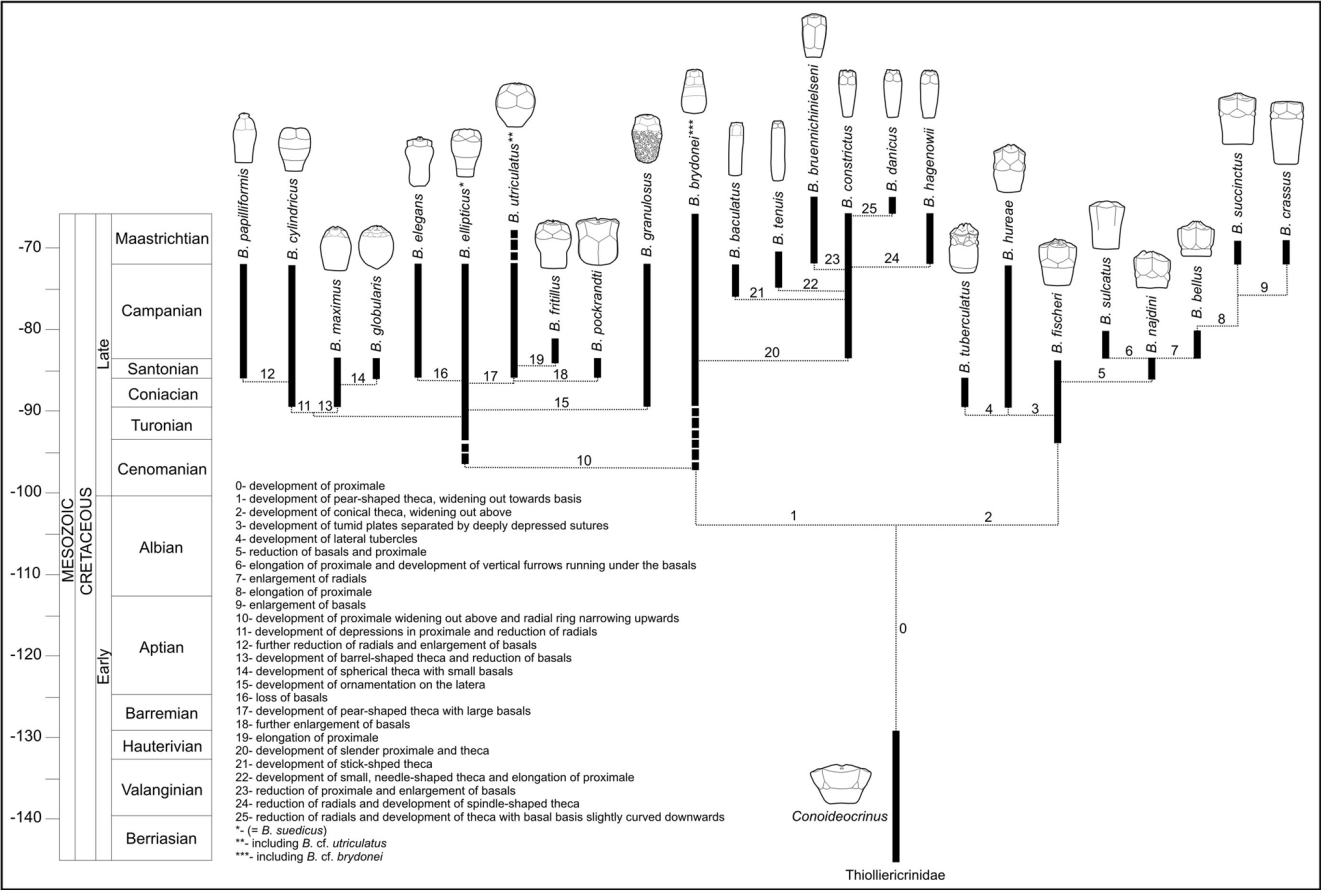
Suggested phylogeny of Cretaceous bourgueticrinid crinoids

Phylogenetic relationships among Cretaceous bourgueticrinid species are controversial because of homoplasy in morphological characters (reversions and parallel evolution, e.g. Kjaer and Thomsen 1999). The simplicity of the morphology of these crinoids is commonly secondary in origin and is not inherited from their true ancestors. Indeed, recent molecular data support that homoplasies are very common in crinoids as a whole, which significantly limit the use of cladistic methods in phylogenetic analyses (Roux et al. 2013). Therefore, in the present paper, preliminary chronostratigraphic analysis was performed to explore possible relationships among Cretaceous bourgueticrinids (Fig. 10).

Bourgueticrinids, classified within the free-living comatulids (Comatulida), are a perfect example of neotenous forms because the adults retained the traits previously seen only in the larval stage (such as the stalk) (Hess and Messing 2011). The Late Cretaceous bourgueticrinid species are thought to be derived from the Early Cretaceous thiolliericrinids (Thiolliericrinidae), which are considered transitional forms between stalkless comatulids and stalked bourgueticrinids because they retained the column distal to the centrodorsal (e.g. Klikushin 1987). In some Early Cretaceous forms, such as *Conoideocrinus*, the disappearance of cirri on the centrodorsal is observed (Klikushin 1987). Thus, these crinoids might be ancestral to the Late Cretacous bourgueticrinids.

In the Cenomanian, divergence of the two main bourgueticrinid lineages occurred (Fig. 10). *Bourgueticrinus* *brydonei* Rasmussen known since the Cenomanian is among the oldest bourgeticrinids (Salamon 2007; Salamon and Gorzelak 2010). This species possesses a pear-shaped theca, widening out towards the basis. This form appears ancestral to two evolutionary lineages, i.e. the *B. constrictus* line, which includes forms with slender proximale and theca, and the *B. ellipticus* line, which includes forms with proximale widening out above and radial ring narrowing upwards. Various morphological forms evolved within the first line, i.e., species with stick-shaped thecae (*B. baculatus*), species with elongated proximale (*B. tenuis*), species with reduced proximale and enlarged basals (*B*. *bruennichinielseni*) and species with reduced radials (*B. danicus*, *B. hagenowii*). Several evolutionary offspring represented by forms with wide, barrel-shaped proximale (*B. cylindricus, B. maximus, B. elegans, B. utriculatus* and *B. granulosus*) also originated from the second line. Among the species with barrel-shaped proximale, the only form displaying tubercular ornamentation on the latera is *B. granulosus*. *B. cylindricus*, in turn, developed depressions in the proximale and a theca with reduced radials. This species appears ancestral to *B. papilliformis,* which is characterized by further reduction of radials and enlarged basals, building a fusiform to claviform theca. *B. maximus* and *B. globularis* with reduced basals and barrel-shaped or spherical theca, respectively, appear to be also closely related to *B. cylindricus*. From the evolutionary line represented by *B. ellipticus*, two additional species likely derived, i.e., *B. utriculatus* with pear-shaped theca displaying large and high basals, and *B. elegans* with completely reduced basals. *B. pockrandti* with enlarged basals and *B. fritillus* with elongated pear-shaped proximale are very closely related and probably descend from *B. utriculatus*.

The second main evolutionary lineage that diverged in the Cenomanian/Turonian is represented by species displaying conical theca widening out above. *B. fischeri* is the oldest species of this lineage. This form possesses low proximale, low and slightly convex basals, and fairly large radials. This form appears to be ancestral to *B. hureae* and *B. tuberculatus*, which displays tumid plates separated by deeply depressed sutures (Fig. 10). In the line leading to *B. crassus*, which also likely descends from *B. fischeri*, significant reversions can be observed. For example, there is a trend to build theca with reduced basals and proximale in the older species (*B. najdini* and *B. bellus*), but this trend is reversed in the younger species (*B. succinctus* and *B. crassus*) (Fig. 10).

## Electronic supplementary material

Below is the link to the electronic supplementary material.

Supplementary material 1 (DOC 570 kb)

## Abbreviations

GIUS: Laboratory of Palaeontology and Stratigraphy of the University of Silesia
GPIH: Geologisch-Paläontologisches Museum in Hamburg
MB: Museum für Naturkunde in Berlin
NHML: Natural History Museum in London
