# Supplementary material for: Late Cretaceous crinoids (Echinodermata) from the southwestern margin of the Holy Cross Mts. (southern Poland) and phylogenetic relationships among bourgueticrinids
Source: Palaontol Z. 2016 Jun 17;90(3):503–20. doi: 10.1007/s12542-016-0313-9 (PMC5448076; doi:10.1007/s12542-016-0313-9)
Supplement: Supplementary file 1 — Supplementary material 1 (DOC 570 kb) [file 12542_2016_313_MOESM1_ESM.doc]

Biometric data of investigated collections. Dimensions of bourgueticrinid cups used in the present study are explained in Fig. 2. Art. f. – articular faces (horiz. – horizontal, sloping – sloping, sl. sl. – slightly sloping), Cup b. – cup base.

GPIH collection (4848-4850)

| **Bhc** | **Bpph** | **Bbph** | **Bbpw** | **h/w** | **Brph** | **Brpw** | **h/w** | **Bwc1** | **Bwc2** | **Bcoh** | **Art. f.** | **Cup b.** |
| --- | --- | --- | --- | --- | --- | --- | --- | --- | --- | --- | --- | --- |
| 7.3 | 3.4 | 1.8 | 3.5 | 0.5 | 2.1 | 3.9 | 0.5 | 6.6 | 7.4 | 1.7 | sl. sl. | elliptical |
| 9 | 4.8 | 2.3 | 3.7 | 0.6 | 1.9 | 3.5 | 0.5 | 5.6 | 6.4 | 1.2 | horiz. | elliptical |
| 7.5 | 3.5 | 2 | 4.4 | 0.5 | 2 | 4.1 | 0.5 | 7.5 | 8.3 | 2.2 | sloping | elliptical |
| 9.4 | 4.8 | 2.6 | 3.7 | 0.7 | 2.1 | 3.3 | 0.6 | 7.4 | 7.4 | 2 | sloping | circular |
| 10.7 | 6 | 2.2 | 3.6 | 0.6 | 2.6 | 3.7 | 0.7 | 7.5 | 7.9 | 2 | sl. sl. | elliptical |
| 9.4 | 4.9 | 2.3 | 3.8 | 0.6 | 2.2 | 3.8 | 0.6 | 6.6 | 7.2 | 1.1 | horiz. | elliptical |
| 9.9 | 5.4 | 2.7 | 4.2 | 0.6 | 1.9 | 4.3 | 0.4 | 6.9 | 8.2 | 1.2 | sl. sl. | elliptical |
| 8.3 | 3.7 | 2.2 | 3.4 | 0.6 | 2.4 | 3.7 | 0.7 | 5.8 | 6.1 | 1.5 | sloping | circular |
| 7.4 | 3.4 | 1.9 | 3.6 | 0.5 | 2.1 | 3.9 | 0.5 | 6.6 | 7.3 | 1.6 | sl. sl. | elliptical |
| 8.1 | 3.8 | 2.3 | 3.7 | 0.6 | 2 | 3.6 | 0.6 | 5.7 | 7 | 1.5 | sl. sl. | elliptical |
| 9.2 | 4.7 | 2.5 | 3.7 | 0.7 | 2 | 3.9 | 0.5 | 6.2 | 6.9 | 1.4 | sloping | elliptical |
| 6.6 | 2.7 | 1.9 | 3.7 | 0.5 | 2 | 4 | 0.5 | 7.2 | 8.4 | 1.8 | sloping | elliptical |
| 7.5 | 4 | 1.8 | 3.3 | 0.5 | 1.6 | 3.6 | 0.5 | 6.9 | 7.4 | 1.1 | sl. sl. | circular |
| 10 | 5.5 | 2.4 | 4 | 0.6 | 2.2 | 4 | 0.5 | 6.6 | 7 | 1.6 | sl. sl. | elliptical |
| 7.5 | 3.3 | 2.3 | 3.8 | 0.6 | 1.9 | 3.6 | 0.5 | 6.5 | 6.9 | 1.5 | sloping | elliptical |
| 8.6 | 3.6 | 2.9 | 4.8 | 0.6 | 2 | 4.5 | 0.5 | 8.2 | 9.1 | 1.9 | sloping | elliptical |
| 7.8 | 3.3 | 2.2 | 4.5 | 0.5 | 2.3 | 4.7 | 0.5 | 7 | 8.2 | 1.3 | sloping | elliptical |
| 6.6 | 3 | 1.9 | 2.8 | 0.7 | 1.8 | 2.7 | 0.7 | 6 | 6.6 | 1.8 | horiz. | elliptical |
| 9.8 | 5.2 | 2.5 | 4.4 | 0.6 | 2.2 | 4 | 0.5 | 6.3 | 7.1 | 1.7 | sl. sl. | circular |
| 6.9 | 3.5 | 1.8 | 3.6 | 0.5 | 1.6 | 3.3 | 0.5 | 6 | 6.5 | 1.4 | horiz. | elliptical |
| 7.3 | 3.4 | 2 | 3.6 | 0.6 | 1.9 | 3.2 | 0.6 | 5.4 | 6.7 | 1.6 | sl. sl. | circular |
| 8.1 | 4.3 | 1.8 | 2.9 | 0.6 | 2 | 2.9 | 0.7 | 6.6 | 8 | 1.2 | sl. sl. | elliptical |
| 8.4 | 3.8 | 2.4 | 4 | 0.6 | 2.3 | 3.9 | 0.6 | 5.9 | 6.6 | 1.5 | horiz. | elliptical |
| 8.3 | 4.2 | 2.1 | 4.5 | 0.5 | 2 | 3.9 | 0.5 | 6.6 | 7.2 | 1.9 | sloping | elliptical |
| 7.3 | 3.1 | 2.2 | 3.9 | 0.6 | 2 | 3.8 | 0.5 | 5.7 | 6.6 | 1.7 | sloping | elliptical |
| 8 | 3.1 | 2.7 | 4.5 | 0.6 | 2.2 | 4 | 0.6 | 6.4 | 7.6 | 1.5 | sloping | circular |
| 6.3 | 1.9 | 2.5 | 4.8 | 0.5 | 1.9 | 3.9 | 0.5 | 6.5 | 7.6 | 1.7 | sloping | circular |
| 7.5 | 2.6 | 2.7 | 4.4 | 0.6 | 2.2 | 3.9 | 0.6 | 7.9 | 8.5 | 1.6 | sloping | elliptical |
| 6.9 | 3.2 | 2 | 3 | 0.7 | 1.8 | 2.6 | 0.7 | 7 | 7.6 | 1.2 | horiz. | elliptical |
| 7.6 | 4 | 1.7 | 3.6 | 0.5 | 1.9 | 3.7 | 0.5 | 6.1 | 6.8 | 1.4 | sl. sl. | circular |
| 8.6 | 4.5 | 2 | 3.3 | 0.6 | 2.1 | 3.4 | 0.6 | 5.6 | 6.6 | 1.9 | sloping | elliptical |
| 7.8 | 3.5 | 2 | 3.7 | 0.5 | 2.3 | 4.1 | 0.6 | 6.2 | 6.8 | 1.1 | sl. sl. | elliptical |
| 9.4 | 4.5 | 2 | 4.4 | 0.5 | 2.8 | 5 | 0.6 | 7.1 | 8.7 | 1.8 | horiz. | elliptical |
| 9.6 | 4.6 | 2 | 4.5 | 0.4 | 2.9 | 4.8 | 0.6 | 8.2 | 9 | 1.7 | sloping | circular |
| 8.3 | 3.7 | 2.2 | 3.7 | 0.6 | 2.4 | 3.4 | 0.7 | 5.8 | 6.1 | 1.2 | sloping | elliptical |
| 7.3 | 3.4 | 1.9 | 3.5 | 0.5 | 2 | 3.9 | 0.5 | 6.6 | 7.2 | 1.6 | sloping | elliptical |
| 8 | 3.8 | 2.3 | 3.7 | 0.6 | 2 | 3.6 | 0.6 | 5.7 | 7 | 1.1 | sloping | elliptical |
| 9.2 | 4.7 | 2.5 | 3.7 | 0.7 | 2 | 3.8 | 0.5 | 6.1 | 6.9 | 1.4 | horiz. | circular |
| 6.5 | 2.6 | 1.9 | 3.7 | 0.5 | 2 | 4 | 0.5 | 7.2 | 8.4 | 1 | sl. sl. | elliptical |
| 7.4 | 4 | 1.8 | 3.2 | 0.6 | 1.6 | 3.6 | 0.5 | 6.9 | 7.4 | 1.8 | sloping | elliptical |
| 10 | 5.4 | 2.4 | 4 | 0.6 | 2.2 | 4 | 0.5 | 6.5 | 7 | 1.5 | horiz. | circular |
| 7.4 | 3.3 | 2.3 | 3.7 | 0.6 | 1.9 | 3.6 | 0.5 | 6.5 | 6.8 | 1.5 | sl. sl. | elliptical |
| 8.6 | 3.6 | 2.9 | 4.8 | 0.6 | 2 | 4.5 | 0.4 | 8.1 | 9 | 1.9 | horiz. | circular |
| 7.7 | 3.2 | 2.2 | 4.5 | 0.5 | 2.3 | 4.7 | 0.5 | 7 | 8.2 | 1.8 | sl. sl. | circular |
| 6.6 | 3 | 1.8 | 2.8 | 0.7 | 1.8 | 2.7 | 0.7 | 6 | 6.6 | 1.1 | sl. sl. | elliptical |
| 9.8 | 5.2 | 2.4 | 4.4 | 0.6 | 2.2 | 4 | 0.5 | 6.3 | 7.1 | 1.7 | sloping | elliptical |
| 6.7 | 3.4 | 1.7 | 3.6 | 0.5 | 1.6 | 3.3 | 0.5 | 6 | 6.5 | 1.5 | sl. sl. | elliptical |
| 7.2 | 3.4 | 2 | 3.6 | 0.6 | 1.9 | 3.2 | 0.6 | 5.4 | 6.7 | 1.2 | sl. sl. | elliptical |
| 8.1 | 4.3 | 1.9 | 2.9 | 0.7 | 1.8 | 2.9 | 0.6 | 6.6 | 8 | 1.6 | sloping | circular |
| 8.4 | 3.8 | 2.3 | 3.9 | 0.6 | 2.4 | 4 | 0.6 | 5.8 | 6.6 | 1.2 | sl. sl. | elliptical |
| 8.2 | 4.2 | 2 | 3.9 | 0.5 | 2 | 4.5 | 0.4 | 6.6 | 7.2 | 1.9 | sloping | elliptical |
| 7.3 | 3.1 | 2.2 | 3.8 | 0.6 | 2 | 3.8 | 0.5 | 5.7 | 6.5 | 1.7 | sl. sl. | elliptical |
| 8 | 3.1 | 2.7 | 4.4 | 0.6 | 2.2 | 3.9 | 0.6 | 6.3 | 7.5 | 1.1 | sl. sl. | circular |
| 5.2 | 1 | 2.4 | 4.7 | 0.5 | 1.8 | 3.9 | 0.5 | 6.4 | 7.2 | 1.6 | sloping | elliptical |
| 7 | 2.4 | 2.7 | 4.4 | 0.6 | 2.1 | 3.8 | 0.6 | 7.9 | 8.5 | 1.6 | horiz. | circular |
| 6.8 | 3.1 | 1.9 | 2.9 | 0.7 | 1.8 | 2.6 | 0.7 | 7 | 7.5 | 1.2 | sl. sl. | elliptical |
| 7.6 | 4 | 1.9 | 3.7 | 0.5 | 1.7 | 3.6 | 0.5 | 6.1 | 6.8 | 1.3 | sloping | elliptical |
| 4.7 | 0.7 | 2.1 | 3.4 | 0.6 | 2 | 3.3 | 0.6 | 5.6 | 6.5 | 1.9 | sloping | circular |
| 6.3 | 2 | 2.3 | 4.1 | 0.6 | 2 | 3.7 | 0.5 | 6.2 | 6.7 | 1.2 | horiz. | elliptical |
| 7.3 | 2.5 | 2 | 4.4 | 0.5 | 2.8 | 5 | 0.6 | 7.1 | 8.7 | 1.8 | sloping | elliptical |
| 4.5 | 0.3 | 2.3 | 3.7 | 0.6 | 1.9 | 3.6 | 0.5 | 6.4 | 7 | 1.6 | sl. sl. | circular |
| 9.6 | 4.6 | 2.8 | 4.8 | 0.6 | 2.1 | 4.5 | 0.5 | 8 | 9.1 | 1.2 | horiz. | circular |
| 8.4 | 3.8 | 2.2 | 3.4 | 0.6 | 2.4 | 3.7 | 0.7 | 5.8 | 6.1 | 1.6 | sl. sl. | elliptical |
| 7.3 | 3.4 | 1.9 | 3.5 | 0.5 | 2 | 3.9 | 0.5 | 6.6 | 7.3 | 1.2 | sloping | elliptical |
| 8.1 | 3.8 | 2 | 3.6 | 0.6 | 2.3 | 3.7 | 0.6 | 5.7 | 7 | 1.6 | sloping | circular |
| 9.3 | 4.7 | 2.1 | 3.7 | 0.6 | 2.5 | 3.9 | 0.7 | 6.1 | 7 | 1.3 | horiz. | elliptical |
| 6.6 | 2.7 | 1.9 | 3.7 | 0.5 | 2 | 4 | 0.5 | 7.2 | 8.4 | 1.8 | sl. sl. | circular |
| 8.8 | 5 | 2 | 3.4 | 0.6 | 1.8 | 3.7 | 0.5 | 6.9 | 7.3 | 1.3 | horiz. | elliptical |
| 10.1 | 5.5 | 2.4 | 4 | 0.6 | 2.2 | 4 | 0.6 | 6.5 | 7 | 1.5 | sloping | elliptical |
| 8.5 | 4.3 | 1.9 | 3.6 | 0.5 | 2.3 | 4 | 0.6 | 6.5 | 6.8 | 1.5 | horiz. | circular |
| 9.5 | 4.6 | 2 | 4.5 | 0.4 | 2.9 | 4.8 | 0.6 | 8.1 | 9 | 1.9 | sloping | circular |
| 8.2 | 3.7 | 2.2 | 4.5 | 0.5 | 2.3 | 4.7 | 0.5 | 7.1 | 8.3 | 1.8 | sl. sl. | elliptical |
| 7.5 | 4 | 1.8 | 2.8 | 0.7 | 1.8 | 2.7 | 0.7 | 6 | 6.7 | 1.8 | sloping | circular |
| 8.6 | 4.2 | 2.3 | 4.3 | 0.5 | 2.1 | 3.9 | 0.5 | 6.3 | 7 | 1.3 | horiz. | circular |
| 5.7 | 2.4 | 1.7 | 3.6 | 0.5 | 1.6 | 3.3 | 0.5 | 6.1 | 6.5 | 1.2 | sloping | elliptical |
| 8.2 | 4.4 | 1.9 | 3.2 | 0.6 | 2 | 3.5 | 0.6 | 5.5 | 6.7 | 1.6 | sl. sl. | elliptical |
| 8.1 | 4.3 | 1.9 | 2.9 | 0.7 | 1.8 | 2.9 | 0.6 | 6.6 | 8 | 1.6 | horiz. | elliptical |
| 8.4 | 3.8 | 2.4 | 4 | 0.6 | 2.3 | 3.9 | 0.6 | 5.9 | 6.7 | 1.5 | horiz. | circular |
| 8.2 | 4.2 | 2 | 4.5 | 0.4 | 2 | 3.9 | 0.5 | 6.7 | 7.2 | 1.5 | sloping | elliptical |
| 8.3 | 4.1 | 2.2 | 3.8 | 0.6 | 2 | 3.8 | 0.5 | 5.7 | 6.5 | 1.7 | sl. sl. | circular |
| 8 | 3.1 | 2.7 | 4.5 | 0.6 | 2.2 | 3.9 | 0.6 | 6.3 | 7.4 | 1.6 | sl. sl. | elliptical |
| 6.3 | 2 | 2.5 | 4.7 | 0.5 | 1.8 | 3.9 | 0.5 | 6.5 | 7.3 | 1.2 | horiz. | circular |
| 8 | 3.4 | 2 | 3.7 | 0.6 | 2.6 | 4.3 | 0.6 | 7.7 | 8.4 | 1.6 | horiz. | elliptical |
| 6.8 | 3.1 | 1.9 | 2.9 | 0.7 | 1.8 | 2.6 | 0.7 | 6.9 | 7.4 | 1.1 | horiz. | elliptical |
| 7.6 | 4 | 1.9 | 3.7 | 0.5 | 1.7 | 3.6 | 0.5 | 6.2 | 6.9 | 1.6 | sl. sl. | elliptical |
| 6.9 | 2.7 | 2.2 | 3.4 | 0.6 | 2 | 3.3 | 0.6 | 5.6 | 6.7 | 1.8 | sloping | circular |
| 9.3 | 5 | 2.3 | 4.1 | 0.6 | 2 | 3.7 | 0.5 | 6.3 | 6.8 | 1.2 | horiz. | circular |
| 7.2 | 2.5 | 2.7 | 4.8 | 0.6 | 2 | 4.3 | 0.5 | 7.1 | 8.6 | 1.1 | sloping | elliptical |
| 7.9 | 3.3 | 2.6 | 4.3 | 0.6 | 2 | 3.7 | 0.5 | 7.8 | 8.3 | 1.6 | sl. sl. | elliptical |
| 8.6 | 3.6 | 2.9 | 4.8 | 0.6 | 2 | 4.5 | 0.5 | 8.1 | 9 | 1.3 | sloping | circular |
| 7.7 | 3.2 | 2.2 | 4.5 | 0.5 | 2.3 | 4.7 | 0.5 | 7.1 | 8.2 | 1.3 | horiz. | circular |
| 7.6 | 4 | 1.9 | 2.8 | 0.7 | 1.8 | 2.7 | 0.7 | 5.9 | 6.6 | 1.1 | sl. sl. | circular |
| 8.6 | 4.2 | 2.4 | 4.3 | 0.5 | 2.1 | 3.9 | 0.5 | 6.4 | 7 | 1.7 | sl. sl. | elliptical |
| 7.9 | 4.5 | 1.8 | 3.6 | 0.5 | 1.6 | 3.3 | 0.5 | 6.1 | 6.7 | 1.1 | sloping | elliptical |
| 7.3 | 3.4 | 2 | 3.6 | 0.6 | 1.9 | 3.2 | 0.6 | 5.4 | 6.7 | 1.6 | sloping | elliptical |
| 8.1 | 4.3 | 2 | 2.9 | 0.7 | 1.8 | 2.9 | 0.6 | 6.6 | 7.9 | 1.6 | sloping | circular |
| 4.5 | 1 | 1.7 | 3.5 | 0.5 | 1.9 | 3.7 | 0.5 | 6.3 | 6.8 | 1.6 | sloping | circular |
| 8.8 | 4.7 | 2.2 | 3.3 | 0.7 | 2 | 3.3 | 0.6 | 6.5 | 6.8 | 1.4 | sl. sl. | elliptical |
| 8.5 | 4.1 | 2 | 3.8 | 0.5 | 2.4 | 4.1 | 0.6 | 6.1 | 6.7 | 1.5 | horiz. | circular |
| 8.6 | 3.5 | 2.2 | 4.4 | 0.5 | 2.9 | 4.9 | 0.6 | 7.3 | 8.6 | 1.8 | horiz. | elliptical |
| 6.7 | 3.1 | 2 | 4.1 | 0.5 | 1.7 | 3.9 | 0.4 | 5.4 | 6.5 | 0.4 | horiz. | circular |
| 8 | 3.5 | 2.5 | 4.2 | 0.6 | 2.1 | 4 | 0.5 | 6.8 | 7.7 | 0.2 | sl. sl. | circular |
| 8.1 | 4.8 | 1.7 | 3.4 | 0.5 | 1.6 | 3.3 | 0.5 | 6 | 7.5 | 0.6 | sloping | elliptical |
| 8 | 3.3 | 2.2 | 4.2 | 0.5 | 2.4 | 4.4 | 0.5 | 7.3 | 8 | 0.6 | sloping | circular |
| 6.7 | 3 | 1.8 | 3.3 | 0.5 | 2 | 3.4 | 0.6 | 6.5 | 7.5 | 0.2 | sloping | elliptical |
| 8.4 | 4.2 | 2.1 | 4 | 0.5 | 2.2 | 4.2 | 0.5 | 6.1 | 7.9 | 0.7 | horiz. | circular |
| 6.1 | 2.2 | 2 | 4 | 0.5 | 1.9 | 3.9 | 0.5 | 6.3 | 7.1 | 0.4 | sloping | elliptical |
| 7 | 3.4 | 1.9 | 3.3 | 0.6 | 1.8 | 3.3 | 0.5 | 4.7 | 5.7 | 0.6 | sl. sl. | circular |
| 7.7 | 3.8 | 2.1 | 4 | 0.5 | 1.8 | 3.8 | 0.5 | 6.2 | 6.5 | 0.5 | sl. sl. | circular |
| 9.2 | 5.3 | 1.8 | 3.6 | 0.5 | 2.1 | 4.2 | 0.5 | 6 | 6.3 | 0.8 | horiz. | elliptical |
| 7.8 | 4.1 | 1.9 | 3.7 | 0.5 | 1.8 | 3.5 | 0.5 | 5.8 | 6.6 | 0.8 | sl. sl. | elliptical |
| 6.7 | 3.3 | 1.8 | 3.6 | 0.5 | 1.7 | 3.5 | 0.5 | 5.6 | 6.5 | 0.3 | sloping | elliptical |
| 7.4 | 2.9 | 2.5 | 4.7 | 0.5 | 2 | 4.3 | 0.5 | 8.4 | 9.3 | 0.6 | horiz. | circular |
| 8.3 | 3.8 | 2.5 | 4.6 | 0.6 | 2 | 4.2 | 0.5 | 7.3 | 7.7 | 0.2 | sl. sl. | circular |
| 8.7 | 4.4 | 2.3 | 4.6 | 0.5 | 2 | 4 | 0.5 | 5.5 | 5.5 | 0.9 | sl. sl. | elliptical |
| 7.1 | 3.4 | 1.7 | 3.9 | 0.4 | 2 | 4.3 | 0.5 | 6.6 | 7.2 | 0.3 | horiz. | circular |
| 6.4 | 1.9 | 2.2 | 4.4 | 0.5 | 2.3 | 4.7 | 0.5 | 7 | 7.2 | 0.7 | sl. sl. | elliptical |
| 7.1 | 3 | 2.2 | 4.3 | 0.5 | 1.9 | 4.2 | 0.5 | 7.8 | 8.5 | 0.2 | horiz. | circular |
| 8.1 | 3.6 | 2.5 | 4 | 0.6 | 2.1 | 3.9 | 0.5 | 6.8 | 7.6 | 0.9 | sloping | elliptical |
| 6.9 | 2.7 | 2 | 4 | 0.5 | 2.2 | 4 | 0.5 | 6.5 | 7 | 0.4 | horiz. | elliptical |
| 6.8 | 2.7 | 2.2 | 4 | 0.5 | 1.9 | 3.8 | 0.5 | 6.4 | 7.1 | 0.3 | sl. sl. | circular |
| 8.2 | 3.8 | 2.3 | 3.6 | 0.6 | 2.1 | 3.2 | 0.6 | 5.9 | 6.5 | 0.2 | horiz. | elliptical |
| 7.3 | 3.4 | 2 | 3.9 | 0.5 | 1.9 | 3.7 | 0.5 | 5.6 | 6.7 | 0.3 | sl. sl. | circular |
| 6.2 | 2.5 | 2.1 | 3.9 | 0.5 | 1.7 | 3.4 | 0.5 | 5.5 | 5.9 | 0.2 | sloping | elliptical |
| 7.8 | 3.3 | 2.4 | 4.2 | 0.6 | 2.1 | 4.1 | 0.5 | 7 | 7.7 | 0.7 | sl. sl. | circular |
| 8.4 | 4.3 | 2 | 3.9 | 0.5 | 2.2 | 4.2 | 0.5 | 6.7 | 7.2 | 0.5 | sl. sl. | circular |
| 7.8 | 3.5 | 2 | 3.9 | 0.5 | 2.3 | 4.2 | 0.5 | 6.1 | 6.6 | 0.3 | sloping | elliptical |
| 6.7 | 2.7 | 1.9 | 3.6 | 0.5 | 2.1 | 3.6 | 0.6 | 6.9 | 7.6 | 0.8 | sl. sl. | circular |
| 7.9 | 3.6 | 2.2 | 4.6 | 0.5 | 2.1 | 4.2 | 0.5 | 7.1 | 8.3 | 0.3 | sl. sl. | elliptical |
| 5.9 | 2.1 | 1.8 | 3.6 | 0.5 | 2 | 3.8 | 0.5 | 6.1 | 6.8 | 0.2 | horiz. | circular |
| 7 | 2.8 | 2.2 | 3.7 | 0.6 | 2 | 3.6 | 0.6 | 6.9 | 7.5 | 0.8 | sloping | elliptical |
| 7.6 | 3.4 | 2.2 | 4 | 0.6 | 2 | 3.7 | 0.5 | 6.7 | 7.4 | 0.3 | sloping | circular |
| 9.7 | 5.4 | 2.2 | 4.3 | 0.5 | 2.1 | 4.1 | 0.5 | 5.7 | 6.9 | 0.7 | sloping | elliptical |
| 9 | 4.7 | 2.1 | 4 | 0.5 | 2.2 | 4.3 | 0.5 | 5.7 | 7.3 | 0.4 | sl. sl. | circular |
| 9.1 | 5 | 2 | 3.7 | 0.5 | 2.1 | 3.9 | 0.5 | 5.1 | 6.8 | 0.2 | horiz. | circular |
| 6.4 | 3.1 | 1.7 | 2.9 | 0.6 | 1.6 | 2.7 | 0.6 | 4.6 | 4.8 | 0.6 | sl. sl. | elliptical |
| 7.9 | 3.6 | 2 | 3.7 | 0.5 | 2.2 | 4 | 0.6 | 5 | 6.1 | 0.8 | horiz. | elliptical |
| 7.4 | 3.6 | 1.8 | 3.2 | 0.6 | 2 | 3.4 | 0.6 | 6.1 | 6.5 | 0.3 | sl. sl. | circular |
| 7.9 | 3.7 | 2.3 | 4 | 0.6 | 2 | 3.9 | 0.5 | 6 | 6.6 | 0.3 | sloping | elliptical |
| 6.3 | 2.6 | 2 | 3.6 | 0.5 | 1.8 | 3.5 | 0.5 | 4.8 | 6.3 | 0.6 | sl. sl. | elliptical |
| 7.1 | 3.3 | 2 | 4.2 | 0.5 | 1.8 | 3.9 | 0.5 | 5.6 | 6.2 | 0.3 | sl. sl. | circular |
| 7.7 | 3.8 | 2 | 4.2 | 0.5 | 1.9 | 4 | 0.5 | 6.2 | 6.8 | 0.9 | sloping | elliptical |
| 7.4 | 3.3 | 1.9 | 4.1 | 0.5 | 2.2 | 4.3 | 0.5 | 6.3 | 6.7 | 0.2 | sloping | elliptical |
| 6.7 | 2.5 | 2 | 4 | 0.5 | 2.1 | 4.2 | 0.5 | 6.3 | 6.7 | 0.3 | sl. sl. | circular |
| 8.1 | 4.1 | 2.1 | 4 | 0.5 | 1.9 | 3.9 | 0.5 | 6.5 | 6.8 | 0.8 | sloping | circular |
| 7.8 | 3.7 | 2.2 | 4 | 0.5 | 2 | 3.7 | 0.5 | 6.2 | 7.1 | 0.2 | sl. sl. | elliptical |
| 5.6 | 3.3 | 2.2 | 4 | 0.6 | 2 | 3.7 | 0.5 | 5.2 | 6.1 | 0.5 | sloping | circular |
| 7.6 | 3.7 | 2 | 3.9 | 0.5 | 1.9 | 3.7 | 0.5 | 5.6 | 6.3 | 0.2 | sl. sl. | circular |
| 7.9 | 4 | 2 | 3.9 | 0.5 | 1.9 | 3.5 | 0.5 | 4.6 | 5.3 | 0.2 | sloping | elliptical |
| 7.7 | 3.5 | 2 | 4 | 0.5 | 2.2 | 4 | 0.5 | 6.4 | 7 | 0.8 | horiz. | circular |
| 7.8 | 3.8 | 1.9 | 3.9 | 0.5 | 2.1 | 4 | 0.5 | 5.5 | 6.1 | 0.3 | sloping | elliptical |
| 7.8 | 3.8 | 2.1 | 4.2 | 0.5 | 1.9 | 3.9 | 0.5 | 6.5 | 6.9 | 0.4 | horiz. | circular |
| 7.6 | 3.7 | 1.9 | 3.8 | 0.5 | 2 | 4 | 0.5 | 6.9 | 7.6 | 0.9 | horiz. | circular |
| 8 | 3.8 | 2 | 3.9 | 0.5 | 2.2 | 4.1 | 0.5 | 4.8 | 5.5 | 0.7 | horiz. | circular |
| 6.6 | 3 | 2 | 4.1 | 0.5 | 1.7 | 3.9 | 0.4 | 5.5 | 6.6 | 0.2 | sl. sl. | elliptical |
| 7.8 | 3.6 | 2.2 | 4.1 | 0.5 | 2 | 3.9 | 0.5 | 6.6 | 7.4 | 0.3 | sl. sl. | circular |
| 8.1 | 4.6 | 1.8 | 3.5 | 0.5 | 1.7 | 3.4 | 0.5 | 5.9 | 7.5 | 0.3 | sl. sl. | elliptical |
| 7.9 | 3.4 | 2.4 | 4.4 | 0.6 | 2.2 | 4.2 | 0.5 | 6.3 | 7 | 0.5 | horiz. | circular |
| 7.6 | 3.9 | 1.8 | 3.9 | 0.4 | 2 | 4.1 | 0.5 | 6.6 | 7.5 | 0.8 | sloping | circular |
| 7.3 | 3.4 | 2 | 4.1 | 0.5 | 1.9 | 3.8 | 0.5 | 6.2 | 7.6 | 0.6 | horiz. | circular |
| 7.1 | 3.2 | 1.9 | 3.9 | 0.5 | 2 | 4 | 0.5 | 6.4 | 7.1 | 0.8 | sloping | elliptical |
| 7.1 | 3.4 | 2 | 3.7 | 0.5 | 1.8 | 3.2 | 0.6 | 4.2 | 5.4 | 0.3 | horiz. | elliptical |
| 7.7 | 3.8 | 2.1 | 4 | 0.5 | 1.8 | 3.8 | 0.5 | 6.3 | 6.9 | 0.3 | sl. sl. | circular |
| 9.3 | 5.4 | 2.1 | 4 | 0.5 | 1.8 | 3.6 | 0.5 | 5.9 | 6.3 | 0.7 | sl. sl. | circular |
| 7.9 | 4.2 | 1.9 | 3.7 | 0.5 | 1.8 | 3.5 | 0.5 | 5.6 | 6.6 | 0.4 | horiz. | circular |
| 6.6 | 3.2 | 1.8 | 3.6 | 0.5 | 1.7 | 3.5 | 0.5 | 5.8 | 6.5 | 0.7 | sl. sl. | elliptical |
| 8.3 | 3.9 | 2.3 | 4.5 | 0.5 | 2.1 | 4.1 | 0.5 | 7.4 | 8.2 | 0.5 | horiz. | circular |
| 8.1 | 3.6 | 2 | 4.1 | 0.5 | 2.5 | 4.6 | 0.6 | 6.3 | 6.7 | 0.2 | sl. sl. | elliptical |
| 8.8 | 4.4 | 2.3 | 4.6 | 0.5 | 2 | 4 | 0.5 | 5.6 | 5.9 | 0.7 | horiz. | circular |
| 7.3 | 3.5 | 2 | 4.3 | 0.5 | 1.8 | 3.9 | 0.5 | 6.7 | 7.2 | 0.4 | sl. sl. | elliptical |
| 7.4 | 2.8 | 2.3 | 4.6 | 0.5 | 2.2 | 4.4 | 0.5 | 7.2 | 7.8 | 0.2 | sloping | circular |
| 7.2 | 3.1 | 1.9 | 4.2 | 0.5 | 2.2 | 4.3 | 0.5 | 6.8 | 7.5 | 0.8 | sl. sl. | elliptical |
| 7.8 | 3.5 | 2.1 | 3.9 | 0.5 | 2.2 | 4 | 0.6 | 6.7 | 7.5 | 0.4 | sl. sl. | circular |
| 7.9 | 3.7 | 2 | 4 | 0.5 | 2.2 | 4 | 0.5 | 6.6 | 7 | 1 | sloping | elliptical |
| 7.8 | 3.7 | 2.2 | 4 | 0.5 | 1.9 | 3.8 | 0.5 | 6.1 | 7.1 | 0.4 | horiz. | elliptical |
| 8.1 | 3.8 | 2.3 | 4 | 0.6 | 2.1 | 3.6 | 0.6 | 5.9 | 6.6 | 0.4 | sl. sl. | circular |
| 7.5 | 3.6 | 2 | 3.9 | 0.5 | 1.9 | 3.7 | 0.5 | 5.5 | 6.5 | 0.5 | sl. sl. | circular |
| 6.9 | 3 | 2.1 | 3.9 | 0.5 | 1.9 | 3.4 | 0.5 | 5.6 | 6 | 0.3 | sl. sl. | circular |
| 7.8 | 3.3 | 2.4 | 4.1 | 0.6 | 2.1 | 4 | 0.5 | 6.9 | 7.6 | 0.5 | sloping | elliptical |
| 7.5 | 3.3 | 2.2 | 4.2 | 0.5 | 2 | 4 | 0.5 | 6.8 | 7.2 | 0.4 | sl. sl. | elliptical |
| 7.7 | 3.5 | 2.2 | 4.2 | 0.5 | 2 | 3.9 | 0.5 | 6.6 | 6.6 | 0.6 | sloping | circular |
| 6.6 | 2.7 | 1.9 | 3.6 | 0.5 | 2 | 3.9 | 0.5 | 7 | 7.6 | 0.2 | sloping | elliptical |
| 7.7 | 3.6 | 2 | 3.9 | 0.5 | 2.1 | 4.1 | 0.5 | 6.1 | 7.2 | 0.9 | horiz. | circular |
| 6.8 | 3.1 | 2 | 3.8 | 0.5 | 1.7 | 3.6 | 0.5 | 6.3 | 6.9 | 0.3 | sl. sl. | elliptical |
| 6.9 | 2.7 | 2 | 3.5 | 0.6 | 2.2 | 3.7 | 0.6 | 6.2 | 7.1 | 0.2 | horiz. | circular |
| 7.4 | 3.4 | 2.1 | 4 | 0.5 | 1.9 | 3.6 | 0.5 | 6.6 | 7.4 | 0.6 | horiz. | elliptical |
| 9.6 | 5.4 | 2.1 | 4.2 | 0.5 | 2 | 4 | 0.5 | 5.2 | 6 | 0.2 | sloping | elliptical |
| 7.7 | 3.6 | 2.1 | 4.1 | 0.5 | 2 | 3.6 | 0.5 | 5.7 | 6.1 | 0.7 | horiz. | circular |
| 7.1 | 3 | 2.1 | 3.9 | 0.5 | 2 | 3.7 | 0.5 | 5.2 | 6.3 | 0.6 | horiz. | elliptical |
| 6.6 | 2.7 | 2 | 4 | 0.5 | 1.9 | 3.7 | 0.5 | 6.2 | 7 | 0.3 | sl. sl. | circular |
| 7.6 | 3.6 | 2 | 4 | 0.5 | 1.9 | 3.6 | 0.5 | 5.2 | 6.5 | 0.3 | sloping | elliptical |
| 6.9 | 3.1 | 2 | 3.5 | 0.6 | 1.8 | 3.3 | 0.6 | 6.7 | 6.9 | 0.4 | horiz. | circular |
| 5.9 | 1.7 | 2 | 3.9 | 0.5 | 2.3 | 4 | 0.6 | 5.5 | 6.1 | 0.2 | sloping | elliptical |
| 6.2 | 2.4 | 2 | 3.6 | 0.6 | 1.8 | 3.5 | 0.5 | 5.8 | 6.3 | 0.6 | sloping | elliptical |
| 7.2 | 3.1 | 2.1 | 4.1 | 0.5 | 2 | 4 | 0.5 | 6.2 | 7 | 0.6 | horiz. | elliptical |
| 7.6 | 3.8 | 2 | 3.6 | 0.6 | 1.8 | 3.3 | 0.5 | 6.4 | 6.9 | 0.3 | horiz | circular |
| 7.3 | 3.2 | 2 | 4 | 0.5 | 2.1 | 4.1 | 0.5 | 6.1 | 7 | 0.3 | sloping | circular |
| 7.2 | 3.3 | 1.9 | 3.8 | 0.5 | 2 | 4 | 0.5 | 6.4 | 7.1 | 0.3 | sloping | elliptical |
| 6.8 | 3.1 | 2 | 3.9 | 0.5 | 1.7 | 3.7 | 0.5 | 5.9 | 6.5 | 0.2 | sl. sl | circular |
| 7.8 | 3.9 | 2.1 | 4 | 0.5 | 1.8 | 3.8 | 0.5 | 6.4 | 6.8 | 0.6 | sloping | elliptical |
| 7.7 | 3.5 | 2.1 | 4 | 0.5 | 2.1 | 3.9 | 0.5 | 7.1 | 8.4 | 0 | sloping | elliptical |
| 8.1 | 3.9 | 2 | 3.8 | 0.5 | 2.1 | 3.9 | 0.5 | 6.5 | 7.7 | 0 | horiz. | circular |
| 7.3 | 3.4 | 2 | 4.1 | 0.5 | 1.9 | 3.9 | 0.5 | 6.6 | 7.9 | 0 | horiz. | circular |
| 7.4 | 3.5 | 2.1 | 4.1 | 0.5 | 1.8 | 3.9 | 0.5 | 7 | 7.9 | 0 | sl. sl. | circular |
| 6.2 | 2.4 | 1.9 | 3.4 | 0.6 | 1.8 | 3.3 | 0.6 | 5.6 | 6.7 | 0 | horiz. | circular |
| 7.8 | 3.9 | 2 | 3.6 | 0.5 | 2 | 3.6 | 0.5 | 6.9 | 7.7 | 0 | sloping | elliptical |
| 7.6 | 3.2 | 2.3 | 4.1 | 0.6 | 2.1 | 4 | 0.5 | 6.9 | 8.2 | 0 | horiz. | circular |
| 8 | 3.8 | 2.2 | 4 | 0.5 | 2 | 4 | 0.5 | 7.2 | 8.7 | 0 | horiz. | circular |
| 8.8 | 4.8 | 2 | 3.9 | 0.5 | 2 | 3.9 | 0.5 | 6 | 7.5 | 0 | sloping | circular |
| 7.2 | 3.4 | 2 | 3.6 | 0.5 | 1.9 | 3.5 | 0.5 | 6.4 | 7.3 | 0 | horiz. | circular |
| 7.3 | 3.2 | 2.1 | 3.9 | 0.5 | 1.9 | 3.9 | 0.5 | 5.8 | 6.8 | 0 | sl. sl. | elliptical |
| 8.8 | 4.6 | 2.2 | 3.7 | 0.6 | 2 | 3.6 | 0.5 | 5.6 | 6.6 | 0 | sl. sl. | elliptical |
| 8 | 3.6 | 2.3 | 4.2 | 0.5 | 2.1 | 4.2 | 0.5 | 7.2 | 8 | 0 | horiz. | circular |
| 7.4 | 3.2 | 2 | 4.1 | 0.5 | 2.2 | 4.2 | 0.5 | 5.5 | 6.2 | 0 | sl. sl. | circular |
| 9.4 | 4.8 | 2.3 | 4.2 | 0.5 | 2.3 | 4.3 | 0.5 | 5.7 | 7 | 0 | horiz. | elliptical |
| 10 | 5.3 | 2.4 | 4.5 | 0.5 | 2.3 | 4.1 | 0.5 | 6.1 | 7.9 | 0 | horiz. | circular |
| 8.1 | 3.8 | 2.1 | 3.9 | 0.5 | 2.2 | 4 | 0.5 | 4.8 | 6 | 0 | sl. sl. | elliptical |
| 7.1 | 3.2 | 2 | 3.8 | 0.5 | 2 | 3.8 | 0.5 | 6 | 7.1 | 0 | horiz. | circular |
| 7.6 | 3.7 | 1.9 | 4 | 0.5 | 2 | 4 | 0.5 | 5.6 | 6.7 | 0 | sl. sl. | elliptical |
| 7.2 | 3 | 2.1 | 4.1 | 0.5 | 2.1 | 4 | 0.5 | 6.4 | 7.1 | 0 | horiz. | circular |
| 7 | 3 | 2.1 | 3.9 | 0.5 | 1.9 | 3.8 | 0.5 | 5.9 | 7 | 0 | horiz. | circular |
| 6.2 | 2.2 | 2.1 | 3.9 | 0.5 | 2 | 3.8 | 0.5 | 5.1 | 6.5 | 0 | sloping | circular |
| 8.7 | 5.2 | 1.8 | 3.4 | 0.5 | 1.7 | 3.2 | 0.5 | 5.9 | 7 | 0 | horiz. | circular |
| 8.1 | 4.5 | 1.9 | 3.5 | 0.5 | 1.8 | 3.4 | 0.5 | 6.4 | 6.9 | 0 | horiz. | elliptical |
| 7.5 | 3.1 | 2.3 | 4.1 | 0.6 | 2.1 | 4 | 0.5 | 6.8 | 7.6 | 0 | sloping | circular |
| 8.4 | 4 | 2.2 | 4 | 0.5 | 2.2 | 4 | 0.6 | 6.5 | 8.1 | 0 | sl. sl. | elliptical |
| 8.8 | 4.5 | 2.3 | 4.1 | 0.6 | 2.1 | 4 | 0.5 | 6.6 | 8.1 | 0 | horiz. | circular |
| 7.8 | 3.8 | 2 | 3.6 | 0.6 | 1.9 | 3.5 | 0.5 | 5.6 | 6.9 | 0 | sloping | circular |
| 8.5 | 4.6 | 1.9 | 3.3 | 0.6 | 2 | 3.5 | 0.6 | 6 | 7 | 0 | horiz. | circular |
| 7.5 | 3.4 | 2 | 3.6 | 0.6 | 2.1 | 3.7 | 0.6 | 6.6 | 7 | 0 | horiz. | circular |
| 7.2 | 3.2 | 2 | 3.8 | 0.5 | 1.9 | 3.6 | 0.5 | 6.4 | 6.8 | 0 | horiz. | elliptical |
| 8.2 | 4.1 | 2 | 4 | 0.5 | 2.1 | 4.1 | 0.5 | 6.2 | 6.8 | 0 | horiz. | circular |
| 7.5 | 3.7 | 1.9 | 3.9 | 0.5 | 2 | 3.9 | 0.5 | 6.1 | 6.9 | 0 | sl. sl. | elliptical |
| 7.7 | 3.7 | 2 | 3.7 | 0.5 | 2 | 3.8 | 0.5 | 6.8 | 7.2 | 0 | horiz. | elliptical |
| 7 | 3.1 | 2 | 3.6 | 0.6 | 1.9 | 3.4 | 0.5 | 6.2 | 7 | 0 | horiz. | circular |
| 8.1 | 4 | 2.1 | 4 | 0.5 | 2 | 3.9 | 0.5 | 6.8 | 7.1 | 0 | sl. sl. | elliptical |
| 6.2 | 2.4 | 1.9 | 3.4 | 0.6 | 1.9 | 3.3 | 0.6 | 5.7 | 6.1 | 0 | horiz. | circular |
| 7.7 | 3.3 | 2.3 | 4.2 | 0.5 | 2.1 | 4.2 | 0.5 | 7.1 | 8.1 | 0 | sloping | circular |
| 6.9 | 3.4 | 1.8 | 3.3 | 0.5 | 1.7 | 3.3 | 0.5 | 5.9 | 6.9 | 0 | horiz. | elliptical |
| 9.1 | 5 | 2.1 | 3.9 | 0.5 | 2 | 3.8 | 0.5 | 6.2 | 7.1 | 0 | horiz. | circular |
| 7.1 | 3.1 | 2 | 3.5 | 0.6 | 2 | 3.6 | 0.6 | 6.1 | 6.9 | 0 | horiz. | elliptical |
| 7.1 | 3.1 | 2 | 3.8 | 0.5 | 2 | 3.7 | 0.5 | 6.3 | 7 | 0 | sloping | circular |
| 7.3 | 3.6 | 1.9 | 3.3 | 0.6 | 1.9 | 3.4 | 0.6 | 6 | 6.9 | 0 | sl. sl. | circular |
| 8.1 | 4 | 2.1 | 4 | 0.5 | 2 | 3.9 | 0.5 | 6.3 | 7 | 0 | horiz. | elliptical |
| 7.4 | 3.2 | 2.1 | 3.9 | 0.5 | 2.2 | 4 | 0.5 | 6.3 | 7.1 | 0 | horiz. | circular |
| 7.1 | 3.2 | 1.9 | 3.3 | 0.6 | 2 | 3.4 | 0.6 | 6.8 | 7.5 | 0 | horiz. | circular |
| 7.6 | 3.5 | 2.1 | 3.8 | 0.5 | 2 | 3.8 | 0.5 | 6.5 | 7.3 | 0 | sloping | elliptical |
| 8.3 | 4.1 | 2.1 | 3.8 | 0.6 | 2.1 | 3.8 | 0.6 | 6.6 | 7.8 | 0 | sl. sl. | circular |
| 7.5 | 3.6 | 1.9 | 3.3 | 0.6 | 2 | 3.4 | 0.6 | 6.3 | 6.9 | 0 | horiz. | circular |
| 7.2 | 3.5 | 1.9 | 3.5 | 0.6 | 1.8 | 3.4 | 0.5 | 6.2 | 6.7 | 0 | horiz. | circular |
| 8.3 | 4.2 | 2 | 4 | 0.5 | 2.1 | 4.1 | 0.5 | 6.6 | 7.3 | 0 | sloping | elliptical |
| 7.2 | 3.2 | 2 | 3.8 | 0.5 | 2 | 3.7 | 0.5 | 5.9 | 6.9 | 0 | sl. sl. | elliptical |
| 7.8 | 4.2 | 1.9 | 3.4 | 0.6 | 1.7 | 3.3 | 0.5 | 4.4 | 5 | 0 | sloping | circular |
| 7.2 | 3.4 | 2 | 3.6 | 0.5 | 1.9 | 3.4 | 0.5 | 5.4 | 6.6 | 0 | sl. sl. | elliptical |
| 7.6 | 3.3 | 2.2 | 4.1 | 0.5 | 2.1 | 4 | 0.5 | 6.6 | 7.2 | 0 | horiz. | circular |
| 8.5 | 4.1 | 2.1 | 4 | 0.5 | 2.2 | 4 | 0.5 | 6.3 | 6.9 | 0 | horiz. | elliptical |
| 7.2 | 3.2 | 2 | 4 | 0.5 | 2.1 | 4 | 0.5 | 6.4 | 6.9 | 0 | horiz. | circular |
| 7.5 | 3.6 | 2 | 3.6 | 0.6 | 1.9 | 3.5 | 0.5 | 5.7 | 6.2 | 0 | sloping | circular |
| 7.4 | 3.5 | 1.9 | 3.3 | 0.6 | 2 | 3.4 | 0.6 | 6.2 | 6.8 | 0 | sloping | elliptical |
| 8.1 | 4.2 | 2 | 4 | 0.5 | 1.9 | 3.9 | 0.5 | 6.4 | 7.3 | 0 | sl. sl. | circular |
| 7.1 | 3.4 | 1.9 | 3.4 | 0.6 | 1.9 | 3.3 | 0.6 | 6.5 | 6.9 | 0 | horiz. | circular |
| 7.6 | 3.4 | 2.1 | 4.1 | 0.5 | 2.1 | 4.1 | 0.5 | 6.1 | 7 | 0 | sloping | elliptical |
| 6.9 | 3.3 | 1.9 | 3.3 | 0.6 | 1.8 | 3.2 | 0.6 | 6.7 | 7.1 | 0 | sl. sl. | circular |
| 8.1 | 4 | 2.1 | 3.9 | 0.5 | 2 | 3.8 | 0.5 | 5.6 | 6.1 | 0 | horiz. | circular |
| 7.5 | 3.7 | 1.9 | 3.9 | 0.5 | 2 | 4 | 0.5 | 6.3 | 7.1 | 0 | sloping | elliptical |
| 7.5 | 3.2 | 2.1 | 4.1 | 0.5 | 2.2 | 4.2 | 0.5 | 6.4 | 7.6 | 0 | sl. sl. | elliptical |
| 7.9 | 3.8 | 2.1 | 3.9 | 0.5 | 2 | 3.8 | 0.5 | 6.4 | 7 | 0 | sloping | circular |
| 7.5 | 3.6 | 2 | 3.9 | 0.5 | 1.9 | 3.7 | 0.5 | 6.6 | 7 | 0 | sl. sl. | circular |
| 7 | 3 | 1.9 | 3.8 | 0.5 | 2 | 3.9 | 0.5 | 6.2 | 6.9 | 0 | horiz. | circular |
| 8.1 | 4.2 | 2 | 3.9 | 0.5 | 1.9 | 3.8 | 0.5 | 6.4 | 6.9 | 0 | sl. sl. | circular |
| 7.7 | 3.7 | 2 | 4 | 0.5 | 2 | 3.9 | 0.5 | 6.1 | 6.7 | 0 | sloping | elliptical |
| 7.3 | 3.3 | 2 | 3.7 | 0.5 | 2 | 3.9 | 0.5 | 6.2 | 6.8 | 0 | horiz. | circular |
| 7.2 | 3.1 | 2 | 3.9 | 0.5 | 2.1 | 3.9 | 0.5 | 5.8 | 6.4 | 0 | sloping | elliptical |
| 7.1 | 3 | 2 | 3.9 | 0.5 | 2.1 | 3.9 | 0.5 | 5.6 | 6.1 | 0 | horiz. | elliptical |
| 7.5 | 3.6 | 2 | 4 | 0.5 | 1.9 | 3.9 | 0.5 | 5.8 | 6.2 | 0 | horiz. | elliptical |
| 7.2 | 3 | 2.1 | 4.1 | 0.5 | 2 | 4 | 0.5 | 6.4 | 7 | 0 | sloping | circular |
| 7.9 | 4 | 2 | 3.9 | 0.5 | 1.9 | 3.8 | 0.5 | 6.8 | 7.3 | 0 | sloping | elliptical |
| 7.3 | 3.3 | 2 | 3.9 | 0.5 | 2 | 4 | 0.5 | 6 | 6.6 | 0 | horiz. | elliptical |
| 7.4 | 3.6 | 1.9 | 3.3 | 0.6 | 2 | 3.5 | 0.6 | 5.6 | 6.7 | 0 | horiz. | circular |
| 7.4 | 3.7 | 1.9 | 3.1 | 0.6 | 1.8 | 3 | 0.6 | 5 | 6.2 | 0 | horiz. | circular |
| 7.7 | 3.2 | 2.3 | 4.1 | 0.6 | 2.2 | 4 | 0.5 | 6.4 | 7.2 | 0 | sl. sl. | circular |
| 8.1 | 4.2 | 1.9 | 3.7 | 0.5 | 2 | 3.7 | 0.5 | 6.6 | 7 | 0 | horiz. | circular |
| 7 | 2.9 | 2.1 | 3.7 | 0.6 | 2 | 3.7 | 0.6 | 6.4 | 6.7 | 0 | sl. sl. | elliptical |
| 7.6 | 3.8 | 1.8 | 3.9 | 0.5 | 2 | 4 | 0.5 | 7.3 | 8 | 0 | horiz. | circular |
| 8.8 | 3.7 | 2.5 | 4.8 | 0.5 | 2.7 | 4.8 | 0.6 | 6.4 | 8.3 | 0 | horiz. | elliptical |
| 6.9 | 2.8 | 2.1 | 3.9 | 0.5 | 2 | 3.8 | 0.5 | 5.4 | 6 | 0 | sl. sl. | circular |
| 7.3 | 3.3 | 2 | 3.5 | 0.6 | 2 | 3.5 | 0.6 | 5.7 | 6.5 | 0 | sloping | circular |
| 7.3 | 3.7 | 1.8 | 3.4 | 0.5 | 1.8 | 3.3 | 0.5 | 4.1 | 5.3 | 0 | sl. sl. | elliptical |
| 8 | 3.6 | 2.2 | 3.8 | 0.6 | 2.2 | 3.8 | 0.6 | 6 | 7.1 | 0 | horiz. | elliptical |
| 8.5 | 4.1 | 2.3 | 4.1 | 0.6 | 2.1 | 4 | 0.5 | 5 | 6.3 | 0 | sloping | elliptical |
| 7.7 | 3.6 | 2.1 | 4 | 0.5 | 2 | 3.9 | 0.5 | 6.1 | 6.9 | 0 | horiz. | circular |
| 7.6 | 3.3 | 2.1 | 3.9 | 0.5 | 2.2 | 3.9 | 0.6 | 5.8 | 6.8 | 0 | sl. sl. | circular |
| 6.2 | 3.1 | 1.6 | 2.9 | 0.5 | 1.5 | 2.9 | 0.5 | 3.8 | 4.4 | 0 | sloping | elliptical |
| 6.7 | 2.5 | 2.1 | 3.8 | 0.6 | 2 | 3.8 | 0.5 | 5.5 | 6.7 | 0 | sloping | elliptical |
| 8 | 3.7 | 2.1 | 3.2 | 0.6 | 2.1 | 3.3 | 0.6 | 4.6 | 5.8 | 0 | horiz. | elliptical |
| 7.1 | 3 | 2 | 3.8 | 0.5 | 2.1 | 3.9 | 0.5 | 6.5 | 7.4 | 0 | horiz. | circular |
| 7.1 | 3 | 2 | 3.9 | 0.5 | 2.1 | 4 | 0.5 | 6 | 7.3 | 0 | sloping | elliptical |
| 8.3 | 4.1 | 2.1 | 4 | 0.5 | 2.1 | 4 | 0.5 | 6.3 | 7 | 0 | sl. sl. | elliptical |
| 7.3 | 3.4 | 2 | 3.8 | 0.5 | 2 | 3.9 | 0.5 | 6.3 | 6.9 | 0 | horiz. | circular |
| 8 | 4 | 2 | 3.7 | 0.5 | 1.9 | 3.6 | 0.5 | 6.2 | 6.9 | 0 | horiz. | circular |

GIUS collection (9-3651/Be)

| **Bhc** | **Bpph** | **Bbph** | **Bbpw** | **h/w** | **Brph** | **Brpw** | **h/w** | **Bwc1** | **Bwc2** | **Bcoh** | **Art. f.** | **Cup b.** |
| --- | --- | --- | --- | --- | --- | --- | --- | --- | --- | --- | --- | --- |
| 9.5 | 3.4 | 3.2 | 4.8 | 0.7 | 2.9 | 4.4 | 0.7 | 7.8 | 8.6 | 1.4 | sloping | elliptical |
| 9 | 4.9 | 2 | 3.4 | 0.6 | 2.1 | 3.6 | 0.6 | 4.8 | 5.9 | 1.3 | horiz. | circular |
| 6.4 | 2.5 | 2.1 | 3.8 | 0.5 | 1.9 | 3.8 | 0.5 | 5.3 | 6.5 | 1 | sl. sl. | elliptical |
| 8.1 | 3.7 | 2.2 | 4.1 | 0.5 | 2.3 | 4.3 | 0.5 | 6.7 | 7.6 | 1.2 | sloping | elliptical |
| 9.2 | 4.6 | 2.2 | 4.5 | 0.5 | 2.5 | 4.7 | 0.5 | 5.6 | 7.6 | 1.2 | sloping | elliptical |
| 9 | 4.9 | 2.1 | 3.7 | 0.6 | 2 | 3.6 | 0.6 | 4.4 | 5.8 | 1.9 | horiz. | circular |
| 8.3 | 4.3 | 2 | 3.6 | 0.6 | 2 | 3.7 | 0.6 | 5.2 | 6 | 1.5 | sl. sl. | circular |
| 7.6 | 3.5 | 2.1 | 3.9 | 0.5 | 2 | 3.9 | 0.5 | 5.5 | 6.4 | 1.1 | sloping | elliptical |
| 7.3 | 3.3 | 2 | 3.9 | 0.5 | 2 | 3.9 | 0.5 | 5.8 | 6.7 | 1.9 | sloping | circular |
| 7.3 | 3.2 | 1.9 | 3.8 | 0.5 | 2.1 | 3.8 | 0.6 | 5.2 | 6.6 | 1.8 | sl. sl. | elliptical |
| 8.7 | 4.3 | 2.2 | 4.4 | 0.5 | 2.3 | 4.5 | 0.5 | 6.5 | 7.5 | 1.3 | sl. sl. | elliptical |
| 7.3 | 3.5 | 1.8 | 3.1 | 0.6 | 2 | 3.2 | 0.6 | 4.7 | 6.5 | 2 | sloping | elliptical |
| 8.9 | 4.6 | 2.3 | 4 | 0.6 | 2 | 3.9 | 0.5 | 6 | 7.6 | 1.8 | sloping | circular |
| 10 | 5 | 2.8 | 4.6 | 0.6 | 2.4 | 4.5 | 0.5 | 7.4 | 8.4 | 2 | horiz. | elliptical |
| 6.7 | 2.7 | 2 | 4.1 | 0.5 | 2 | 4.1 | 0.5 | 6.6 | 7.7 | 1.8 | sloping | circular |
| 8.8 | 3.9 | 2.3 | 4 | 0.6 | 2.7 | 4.1 | 0.7 | 6.4 | 7.6 | 1.3 | horiz. | elliptical |
| 7.4 | 3.3 | 2.2 | 4.1 | 0.5 | 2 | 4 | 0.5 | 5.4 | 6.5 | 1.8 | sloping | elliptical |
| 7.8 | 2.8 | 2.7 | 4.4 | 0.6 | 2.3 | 4.1 | 0.6 | 6 | 7.1 | 0.6 | sloping | elliptical |
| 7.4 | 3.2 | 2.1 | 3.8 | 0.6 | 2.2 | 3.8 | 0.6 | 5.7 | 6.4 | 0.7 | horiz. | elliptical |
| 7.7 | 3.5 | 2 | 4 | 0.5 | 2.2 | 4.1 | 0.5 | 5.1 | 6.1 | 0.5 | horiz. | circular |
| 8.2 | 4.5 | 1.9 | 3.9 | 0.4 | 1.8 | 3.9 | 0.5 | 5.9 | 7 | 0.8 | sloping | elliptical |
| 6.6 | 2.9 | 1.9 | 3.1 | 0.6 | 1.8 | 3 | 0.6 | 4.7 | 5.2 | 0.2 | sl. sl. | circular |
| 9.7 | 5.1 | 2.2 | 4 | 0.5 | 2.4 | 4.1 | 0.6 | 5.3 | 7 | 0.2 | horiz. | circular |
| 8.8 | 4.1 | 2.5 | 4.2 | 0.6 | 2.2 | 4.1 | 0.5 | 6.6 | 7.7 | 0.5 | sloping | elliptical |
| 8.4 | 4 | 2.2 | 4.1 | 0.5 | 2.2 | 4.2 | 0.5 | 6.8 | 7.3 | 0.2 | sl. sl. | elliptical |
| 7.7 | 3.9 | 2 | 3.7 | 0.5 | 1.8 | 3.6 | 0.5 | 5 | 5.8 | 0 | horiz. | circular |
| 7.8 | 4.1 | 1.8 | 3.4 | 0.5 | 1.9 | 3.6 | 0.5 | 6.1 | 6.7 | 0 | sloping | circular |
| 8.5 | 4.8 | 1.8 | 3.8 | 0.5 | 2 | 3.9 | 0.5 | 6.1 | 7.5 | 0 | horiz. | elliptical |
| 8.8 | 5.2 | 1.7 | 3.2 | 0.5 | 1.8 | 3.2 | 0.6 | 6.8 | 7.9 | 0 | sloping | elliptical |
| 7.3 | 3.4 | 2 | 4 | 0.5 | 1.9 | 3.9 | 0.5 | 6.3 | 7.2 | 0 | horiz. | circular |
| 7.4 | 3.3 | 2.1 | 4.1 | 0.5 | 2 | 4 | 0.5 | 6.6 | 7.2 | 0 | sl. sl. | circular |
| 7.1 | 3.5 | 1.8 | 3.5 | 0.5 | 1.8 | 3.6 | 0.5 | 6.1 | 6.6 | 0 | sloping | elliptical |
| 8.2 | 4.5 | 1.9 | 4 | 0.5 | 1.8 | 4 | 0.5 | 8.4 | 8.9 | 0 | horiz. | circular |
| 6.5 | 2.4 | 2 | 3.9 | 0.5 | 2 | 4 | 0.5 | 6.2 | 7 | 0 | horiz. | elliptical |
| 8 | 3.8 | 2.1 | 4.1 | 0.5 | 2.1 | 4 | 0.5 | 6.6 | 7.7 | 0 | horiz. | circular |
| 7.9 | 3.6 | 2.1 | 4 | 0.5 | 2.2 | 4.2 | 0.5 | 6 | 7.3 | 0 | horiz. | elliptical |
| 7.1 | 2.9 | 2.2 | 4.1 | 0.5 | 2.1 | 4.1 | 0.5 | 6.4 | 7.2 | 0 | sloping | circular |

NHML collection (E 45168-86, E 45320-6)

| **Bhc** | **Bpph** | **Bbph** | **Bbpw** | **h/w** | **Brph** | **Brpw** | **h/w** | **Bwc1** | **Bwc2** | **Bcoh** | **Art. f.** | **Cup b.** |
| --- | --- | --- | --- | --- | --- | --- | --- | --- | --- | --- | --- | --- |
| 8.1 | 4.1 | 2.1 | 4.2 | 0.5 | 2 | 4 | 0.5 | 5.8 | 7.2 | 0 | horiz. | elliptical |
| 8.5 | 4.5 | 2 | 3.9 | 0.5 | 2 | 3.9 | 0.5 | 5.4 | 6.8 | 0 | horiz. | elliptical |
| 7.5 | 3.3 | 2.2 | 3.9 | 0.6 | 2 | 3.8 | 0.5 | 7.3 | 8 | 0 | horiz. | circular |
| 9.4 | 5.5 | 1.9 | 3.9 | 0.5 | 2 | 4 | 0.5 | 6 | 6.5 | 0 | horiz. | elliptical |
| 7.4 | 3.1 | 2 | 4.1 | 0.5 | 2.3 | 4.7 | 0.5 | 7.1 | 7.8 | 0 | horiz. | elliptical |
| 7.2 | 3.2 | 2.1 | 4 | 0.5 | 1.9 | 3.7 | 0.5 | 5.8 | 6.4 | 0 | horiz. | circular |
| 6.5 | 3.4 | 1.6 | 3.6 | 0.4 | 1.5 | 3.5 | 0.4 | 5.9 | 6.8 | 0 | horiz. | circular |
| 9.2 | 3.8 | 2.9 | 4.9 | 0.6 | 2.5 | 4.3 | 0.6 | 7.2 | 8.1 | 0 | horiz. | circular |
| 8.3 | 4.4 | 2.1 | 3.8 | 0.6 | 1.8 | 3.1 | 0.6 | 6 | 6.5 | 0 | horiz. | elliptical |
| 8.4 | 4.2 | 2.2 | 4 | 0.5 | 2 | 3.9 | 0.5 | 6 | 6.9 | 0 | horiz. | elliptical |
| 8.2 | 2.1 | 3.2 | 5.1 | 0.6 | 3 | 5 | 0.6 | 7 | 8.3 | 0 | horiz. | circular |
| 12.8 | 7.2 | 2.8 | 5 | 0.6 | 2.8 | 4.9 | 0.6 | 7.8 | 9.1 | 0 | horiz. | elliptical |
| 9.6 | 5 | 2.3 | 4.5 | 0.5 | 2.2 | 4.4 | 0.5 | 6.9 | 7.3 | 0 | horiz. | circular |
| 9.9 | 5.7 | 2.1 | 3.9 | 0.5 | 2.1 | 3.9 | 0.5 | 6.3 | 7.2 | 0 | horiz. | elliptical |
| 8 | 4.1 | 2 | 3.3 | 0.6 | 2 | 3.3 | 0.6 | 5.2 | 5.9 | 0 | horiz. | elliptical |
| 6.1 | 2.2 | 2 | 3.4 | 0.6 | 1.9 | 3.4 | 0.6 | 4.9 | 5.2 | 0 | horiz. | circular |
| 8.2 | 4.5 | 1.8 | 3.3 | 0.5 | 1.9 | 3.4 | 0.6 | 5.2 | 5.8 | 0 | horiz. | elliptical |
| 6.8 | 3 | 1.9 | 3.8 | 0.5 | 1.9 | 3.8 | 0.5 | 5.3 | 6.2 | 0 | horiz. | elliptical |
| 7.6 | 3.3 | 2.2 | 4.3 | 0.5 | 2.1 | 4 | 0.5 | 6.5 | 7.2 | 0 | horiz. | circular |
| 5.4 | 2.1 | 1.7 | 3.4 | 0.5 | 1.6 | 3.3 | 0.5 | 4.2 | 5.2 | 0 | horiz. | circular |
| 8.6 | 4.9 | 1.8 | 3.5 | 0.5 | 1.9 | 3.7 | 0.5 | 6 | 7.2 | 0 | horiz. | circular |
| 4.5 | 1.9 | 1.3 | 3 | 0.4 | 1.3 | 3.1 | 0.4 | 5.1 | 6.2 | 0 | horiz. | circular |
| 6.3 | 3.2 | 1.6 | 2.9 | 0.6 | 1.5 | 2.7 | 0.6 | 3.9 | 4.3 | 0 | horiz. | circular |
| 7.5 | 4.1 | 1.7 | 3.3 | 0.5 | 1.7 | 3.4 | 0.5 | 5.3 | 5.7 | 0 | horiz. | elliptical |
| 7.3 | 3.3 | 2 | 3.7 | 0.5 | 2 | 3.9 | 0.5 | 6.2 | 6.8 | 0 | horiz. | elliptical |
| 7.3 | 3.2 | 2 | 3.9 | 0.5 | 2.1 | 3.9 | 0.5 | 5.8 | 6.4 | 0 | horiz. | circular |
| 7.2 | 3.1 | 2 | 4 | 0.5 | 2.1 | 4.1 | 0.5 | 5.7 | 6 | 0 | horiz. | circular |
| 7.1 | 3 | 2 | 4.2 | 0.5 | 2.1 | 4.4 | 0.5 | 6.9 | 7.6 | 0 | horiz. | elliptical |
| 7.5 | 3.3 | 2.1 | 4 | 0.5 | 2.1 | 4 | 0.5 | 6.3 | 7.2 | 0 | horiz. | elliptical |
| 6.1 | 2.2 | 2 | 3.7 | 0.5 | 2 | 3.6 | 0.5 | 5.3 | 5.9 | 0 | horiz. | circular |
| 6.1 | 2.2 | 2 | 3.9 | 0.5 | 1.9 | 3.8 | 0.5 | 4.9 | 5.6 | 0 | horiz. | elliptical |
| 7.2 | 3.4 | 1.9 | 3.3 | 0.6 | 1.9 | 3.3 | 0.6 | 5.2 | 5.8 | 0 | horiz. | elliptical |
| 6.9 | 3.1 | 1.9 | 3.8 | 0.5 | 1.9 | 3.9 | 0.5 | 5.2 | 5 | 0 | horiz. | elliptical |
| 7.1 | 3 | 2.1 | 4 | 0.5 | 2 | 3.9 | 0.5 | 6.3 | 7 | 0 | horiz. | elliptical |
| 6.4 | 2.1 | 2.1 | 3.9 | 0.5 | 2.2 | 4 | 0.5 | 6.3 | 7 | 0 | horiz. | circular |
| 7 | 3.1 | 1.9 | 3.3 | 0.6 | 2 | 3.4 | 0.6 | 6.8 | 7.6 | 0 | horiz. | elliptical |
| 7.2 | 3.1 | 2.1 | 3.8 | 0.5 | 2 | 3.8 | 0.5 | 6.5 | 7 | 0 | horiz. | elliptical |
| 7.1 | 3.2 | 1.9 | 3.3 | 0.6 | 2 | 3.4 | 0.6 | 6.8 | 7.6 | 0 | horiz. | circular |
| 7.6 | 3.6 | 2 | 3.8 | 0.5 | 2 | 3.7 | 0.5 | 6.4 | 7.2 | 0 | horiz. | circular |
| 6.3 | 2.1 | 2.1 | 3.8 | 0.6 | 2.2 | 3.9 | 0.6 | 6.7 | 7.8 | 0 | horiz. | circular |
| 8.5 | 4.6 | 1.9 | 3.3 | 0.6 | 2 | 3.5 | 0.6 | 6.4 | 7 | 0 | horiz. | circular |
| 7.1 | 3.4 | 1.9 | 3.4 | 0.6 | 1.8 | 3.4 | 0.5 | 6.1 | 6.8 | 0 | horiz. | elliptical |
| 8.3 | 4.1 | 2 | 4 | 0.5 | 2.1 | 4.1 | 0.5 | 6.6 | 7.2 | 0 | horiz. | circular |
| 7.2 | 3.2 | 2 | 3.7 | 0.5 | 2 | 3.7 | 0.5 | 5.8 | 6.4 | 0 | horiz. | circular |
| 6.4 | 3.1 | 1.7 | 2.9 | 0.6 | 1.6 | 2.8 | 0.6 | 4.6 | 4.8 | 0 | horiz. | elliptical |
| 7.7 | 3.5 | 2 | 4 | 0.5 | 2.2 | 4.1 | 0.5 | 5 | 6 | 0 | horiz. | elliptical |
| 7.6 | 3.5 | 2.1 | 4 | 0.5 | 2 | 3.8 | 0.5 | 6.5 | 6.9 | 0 | horiz. | circular |
| 6.5 | 2.2 | 2.2 | 4.2 | 0.5 | 2.1 | 4.1 | 0.5 | 7 | 7.6 | 0 | horiz. | elliptical |
| 8.3 | 4.2 | 2 | 3.9 | 0.5 | 2.1 | 4.1 | 0.5 | 6.8 | 7.2 | 0 | horiz. | elliptical |
| 6.7 | 2.6 | 2 | 3.9 | 0.5 | 2.1 | 4 | 0.5 | 6.2 | 6.7 | 0 | horiz. | circular |
| 5.7 | 1.7 | 1.9 | 3.6 | 0.5 | 2.1 | 3.6 | 0.6 | 6.9 | 7.4 | 0 | horiz. | circular |
| 8 | 3.7 | 2.2 | 4.2 | 0.5 | 2.1 | 4.1 | 0.5 | 7.1 | 7.7 | 0 | horiz. | circular |
| 6 | 2.1 | 1.9 | 3.8 | 0.5 | 2 | 3.8 | 0.5 | 6 | 6.7 | 0 | horiz. | elliptical |
| 6.9 | 2.7 | 2.1 | 3.8 | 0.6 | 2 | 3.7 | 0.6 | 6.8 | 7.2 | 0 | horiz. | elliptical |
| 7.5 | 3.3 | 2.2 | 4 | 0.6 | 2 | 3.7 | 0.5 | 6.8 | 7.1 | 0 | horiz. | elliptical |
| 7.6 | 3.4 | 2.2 | 4.2 | 0.5 | 2.1 | 4.1 | 0.5 | 5.7 | 6.3 | 0 | horiz. | circular |
| 6.3 | 2 | 2.1 | 3.9 | 0.5 | 2.2 | 4.1 | 0.5 | 5.7 | 6.8 | 0 | horiz. | circular |
| 6.5 | 2.4 | 2 | 3.7 | 0.5 | 2 | 3.8 | 0.5 | 5.1 | 6 | 0 | horiz. | circular |
| 6.4 | 3.1 | 1.7 | 2.9 | 0.6 | 1.6 | 2.8 | 0.6 | 4.6 | 4.8 | 0 | horiz. | elliptical |
| 6.8 | 2.6 | 2 | 3.7 | 0.5 | 2.2 | 4 | 0.5 | 5 | 6 | 0 | horiz. | circular |
| 7.5 | 3.6 | 1.9 | 3.3 | 0.6 | 2 | 3.4 | 0.6 | 6.2 | 6.6 | 0 | horiz. | circular |
| 7.1 | 3.1 | 2 | 4 | 0.5 | 2 | 4 | 0.5 | 6 | 6.6 | 0 | horiz. | elliptical |
| 5.9 | 2.1 | 2 | 3.6 | 0.5 | 1.9 | 3.5 | 0.5 | 4.8 | 5.3 | 0 | horiz. | elliptical |
| 8.3 | 4.3 | 2 | 4.1 | 0.5 | 2 | 4 | 0.5 | 5.6 | 6.3 | 0 | horiz. | elliptical |
| 8.8 | 4.8 | 2 | 4 | 0.5 | 2 | 4 | 0.5 | 6.3 | 7 | 0 | horiz. | circular |
| 7.4 | 3.2 | 2 | 4 | 0.5 | 2.2 | 4.3 | 0.5 | 6.5 | 7 | 0 | horiz. | circular |
| 7.5 | 3.5 | 2 | 4 | 0.5 | 2 | 4 | 0.5 | 6.3 | 6.8 | 0 | horiz. | elliptical |
| 6.1 | 2.1 | 2.1 | 4 | 0.5 | 1.9 | 3.8 | 0.5 | 6.6 | 6.6 | 0 | horiz. | elliptical |
| 7.7 | 3.6 | 2.1 | 4 | 0.5 | 2 | 3.9 | 0.5 | 6.3 | 7 | 0 | horiz. | circular |
| 7.5 | 3.2 | 2.2 | 4.1 | 0.5 | 2.1 | 4 | 0.5 | 5.3 | 6.2 | 0 | horiz. | elliptical |
| 7.5 | 3.6 | 2 | 3.9 | 0.5 | 1.9 | 3.8 | 0.5 | 5.6 | 6.3 | 0 | horiz. | elliptical |
| 6.9 | 2.9 | 2 | 4 | 0.5 | 2 | 3.9 | 0.5 | 4.6 | 5 | 0 | horiz. | circular |
| 7.2 | 3.4 | 2 | 3.6 | 0.5 | 1.9 | 3.5 | 0.5 | 6.4 | 6.8 | 0 | horiz. | elliptical |
| 7.4 | 3.3 | 2.1 | 3.9 | 0.5 | 2 | 3.8 | 0.5 | 5.8 | 6.4 | 0 | horiz. | circular |
| 6.7 | 2.6 | 2.1 | 3.9 | 0.5 | 2 | 3.7 | 0.5 | 5.7 | 6.3 | 0 | horiz. | elliptical |
| 6.7 | 2.6 | 2 | 4 | 0.5 | 2.1 | 4.1 | 0.5 | 6.2 | 7 | 0 | horiz. | elliptical |
| 7.3 | 3.2 | 2 | 4.1 | 0.5 | 2.1 | 4.2 | 0.5 | 5.5 | 6.2 | 0 | horiz. | circular |
| 9.2 | 4.7 | 2.2 | 4.2 | 0.5 | 2.2 | 4.3 | 0.5 | 5.6 | 6.9 | 0 | horiz. | circular |
| 7.6 | 3.4 | 2.1 | 4 | 0.5 | 2.1 | 4.1 | 0.5 | 6.6 | 7.1 | 0 | horiz. | elliptical |
| 5.9 | 2.2 | 1.9 | 3.3 | 0.6 | 1.8 | 3.2 | 0.6 | 6.8 | 7.1 | 0 | horiz. | circular |
| 6.9 | 2.8 | 2.1 | 3.9 | 0.5 | 2 | 3.8 | 0.5 | 5.5 | 6 | 0 | horiz. | elliptical |
| 6.9 | 3.1 | 1.9 | 3.9 | 0.5 | 2 | 3.9 | 0.5 | 6 | 7 | 0 | horiz. | elliptical |
| 7.1 | 3 | 2 | 4.1 | 0.5 | 2.1 | 4.2 | 0.5 | 6.3 | 7.2 | 0 | horiz. | circular |
| 6.9 | 2.8 | 2.1 | 3.9 | 0.5 | 2 | 3.8 | 0.5 | 6.4 | 7 | 0 | horiz. | elliptical |
| 7.5 | 3.5 | 2.1 | 3.9 | 0.5 | 1.9 | 3.8 | 0.5 | 6.6 | 6.9 | 0 | horiz. | circular |
| 6.1 | 2.1 | 2 | 3.8 | 0.5 | 2 | 3.9 | 0.5 | 6 | 6.8 | 0 | horiz. | elliptical |
| 7.2 | 3.2 | 2 | 3.9 | 0.5 | 1.9 | 3.8 | 0.5 | 6.4 | 6.9 | 0 | horiz. | circular |
| 7.7 | 3.7 | 2 | 4 | 0.5 | 2 | 3.9 | 0.5 | 6.1 | 6.8 | 0 | horiz. | circular |
| 6.2 | 2.2 | 2 | 3.7 | 0.5 | 2 | 3.9 | 0.5 | 6.1 | 6.8 | 0 | horiz. | elliptical |
| 7.2 | 3.1 | 2 | 3.9 | 0.5 | 2.1 | 4 | 0.5 | 6 | 6.5 | 0 | horiz. | elliptical |
| 6.5 | 2.3 | 2 | 3.9 | 0.5 | 2.1 | 3.9 | 0.5 | 5.7 | 6.1 | 0 | horiz. | circular |
| 7.5 | 3.6 | 2 | 4 | 0.5 | 1.9 | 3.9 | 0.5 | 5.9 | 6.3 | 0 | horiz. | circular |
| 7.2 | 3 | 2.1 | 4.1 | 0.5 | 2.1 | 4 | 0.5 | 6.4 | 7.1 | 0 | horiz. | circular |
| 7.1 | 3 | 2.1 | 4 | 0.5 | 2 | 3.8 | 0.5 | 6.9 | 7.4 | 0 | horiz. | elliptical |
| 6.9 | 3 | 2 | 4 | 0.5 | 1.9 | 3.9 | 0.5 | 6.4 | 7.2 | 0 | horiz. | circular |
| 6.7 | 2.5 | 2 | 3.9 | 0.5 | 2.2 | 4.1 | 0.5 | 7 | 7.9 | 0 | horiz. | circular |
| 6.8 | 3.1 | 1.9 | 3.9 | 0.5 | 1.8 | 3.8 | 0.5 | 6.6 | 7.1 | 0 | horiz. | elliptical |
| 6.8 | 3.1 | 1.9 | 3.8 | 0.5 | 1.8 | 3.7 | 0.5 | 6.2 | 6.9 | 0 | horiz. | elliptical |
| 6.6 | 2.6 | 2.1 | 4 | 0.5 | 2 | 3.9 | 0.5 | 6.5 | 7 | 0 | horiz. | circular |
| 7.4 | 3.4 | 2 | 4 | 0.5 | 2 | 4 | 0.5 | 6.3 | 6.9 | 0 | horiz. | circular |
